# XEOL and Persistent Luminescence in Eu- and Ti-Doped Lu_2_O_2_S Materials

**DOI:** 10.1021/acsomega.5c03406

**Published:** 2025-07-22

**Authors:** Karina T. Fonseca, Nataly S. Santos, Marcelo C. Portes, Fernando A. Garcia, Lucas C. V. Rodrigues

**Affiliations:** † Department of Fundamental Chemistry, Institute of Chemistry, 28133 University of São Paulo, São Paulo-SP 05508-000, Brazil; ‡ Department of Applied Physics, Institute of Physics, University of São Paulo, São Paulo-SP 05508-900, Brazil

## Abstract

Rare-earth oxysulfides (RE_2_O_2_S, RE^3+^ = Y, La, Gd, Lu) are promising matrices for luminescent materials due to their high thermal and chemical stability, cost-effectiveness, and efficient sensitization of trivalent lanthanide ions, leading to high luminescent efficiency. These compounds crystallize in a trigonal structure, belonging to the space group *P*3̅*m*1. Lutetium oxysulfide (Lu_2_O_2_S) has been extensively studied as a host material for three-dimensional plasma display panels, field emission displays, and light-emitting diodes. Lu_2_O_2_S:Eu^3+^ exhibits red persistent luminescence, while Lu_2_O_2_S:Ti features a broad orange emission band associated with titanium. The incorporation of Mg^2+^ enhances afterglow duration by creating charge compensation defects and facilitating energy storage in trap levels. This work investigates the crystalline structure, optical absorption, and persistent luminescence properties of Lu_2_O_2_S and its doped variants: Lu_2_O_2_S:Eu^3+^, Lu_2_O_2_S:Ti, and Lu_2_O_2_S:Mg^2+^. Additionally, codoping effects were explored in Lu_2_O_2_S:Eu^3+^,Ti, Lu_2_O_2_S:Eu^3+^,Mg^2+^, Lu_2_O_2_S:Ti,Mg^2+^, and Lu_2_O_2_S:Eu^3+^,Ti,Mg^2+^. The materials were synthesized for the first time via a rapid and energy-efficient microwave-assisted solid-state method. Phase purity and crystal structure were analyzed by X-ray diffraction (XRD) with Rietveld refinement. The incorporation of Eu^3+^, Ti and Mg^2+^ was assessed along with resulting structural modifications. Lu_2_O_2_S band gap energy was obtained with Kubelka–Munk function on the diffuse reflectance spectroscopy (DRS) data. X-ray Absorption Near Edge Structure (XANES) and X-ray Excited Optical Luminescence (XEOL) measurements confirmed that absorption by the matrix at the Lu L_3_-edge effectively induces luminescence, playing a positive role in the emission mechanism. EPR spectra of Lu_2_O_2_S:Ti and Lu_2_O_2_S:Eu^3+^,Ti materials suggested that, even though Ti^3+^ might be present, photoredox processes are absent in the persistent luminescence mechanism and that Ti remains in the Ti^4+^ state. The observed visible-light emissions upon UV and X-ray excitation, along with the high energy storage capacity, highlight the potential of these materials for applications in dosimetry, bioimaging, and optoelectronic devices.

## Graphical abstract

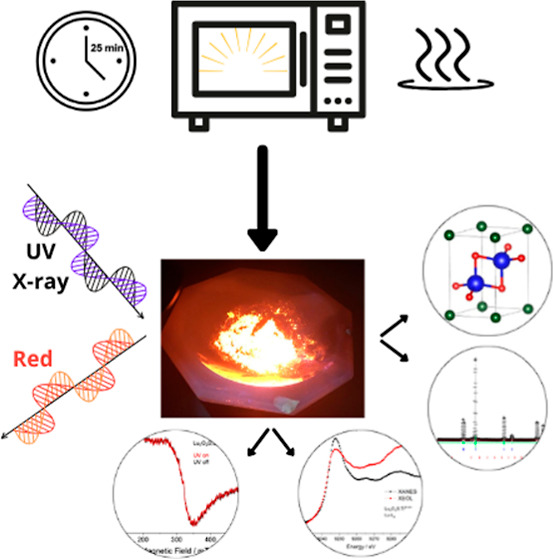

## 1 Introduction

Rare-earth oxysulfides (RE_2_O_2_S, RE^3+^ = Y, La, Gd, Lu) have been extensively studied as host materials for luminescent applications due to their high thermal and chemical stability, remarkable optical properties, and relatively low production cost. These compounds adopt a trigonal structure and crystallize in the space group *P*3̅*m*1, which supports a layered arrangement of oxygen and sulfur anions around the rare-earth cations. This structural configuration contributes to their excellent ability to accommodate various activator ions without significant lattice distortion, making them highly versatile for different photonic applications, related to cathodoluminescence, persistent luminescence, upconversion, and scintillation.^1–4^ Scintillators based on RE_2_O_2_S have been extensively studied for indirect X-ray detection in imaging screens, driving progress in both medical diagnostics and industrial imaging technologies. Among them, lutetium oxysulfide (Lu_2_O_2_S) stands out as a particularly attractive candidate due to its dense crystal structure and potential scintillation properties, making it a key material in radiation detection. As a dense, high-Z material with low optical absorption, Lu_2_O_2_S can efficiently convert high-energy radiation into visible light, making it suitable for X-ray imaging, medical diagnostics, and security screening.

The incorporation of dopants such as Eu^3+^ enhances its scintillation efficiency, enabling higher sensitivity in detection applications. The choice of activator ions is fundamental in tuning emission properties for desired applications. For instance, Eu^3+^ emits in the visible region, leading to intense red luminescence; Ti^3+^ or Ti^4+^ may generate broad emissions in the orange range, while codoping with Mg^2+^ creates charge compensation sites and additional traps that extend the afterglow duration. Moreover, activators like Nd^3+^ and Yb^3+^ can be employed to access emissions in the near-infrared (NIR) region, which is crucial for bioimaging and telecommunications. UV emissions are also of interest for sterilization and analytical applications, while visible emissions are essential for display and lighting technologies.

Several lutetium-based scintillators have garnered significant attention in recent years due to the pursuit of more efficient materials for applications in medical imaging involving high-energy spectroscopy. Cerium-doped lutetium oxyorthosilicate (Lu_2_SiO_5_:Ce, or LSO:Ce) and lutetium yttrium oxyorthosilicate (Lu_2(1–*x*)_Y_2*x*_SiO_5_:Ce, or LYSO:Ce) are currently the most commonly utilized scintillator materials in positron emission tomography (PET) detectors.^5–8^ Their popularity stems from their advantageous physical characteristics for detecting 511 keV annihilation photons, including high light yield, a substantial linear attenuation coefficient, and a fast decay time.^9–11^ Other lutetium-based inorganic scintillators, such as LuAlO_3_:Ce, Lu_2_Si_2_O_7_:Ce, and Lu_2_O_3_:Eu, have also been explored as potential candidates for nuclear medicine detection systems.^12–15^ Additional lutetium-based materials include transparent ceramic scintillators, which are fabricated from nanoscale garnet powders that undergo a multistage process involving sintering and annealing to achieve a transparent ceramic structure. Lanthanide gallium/aluminum-based garnets, such as LuAG:Ce and GLuGAG:Ce, stand out as the most promising due to their high transparency and impressive light yield of approximately 60,000 photons per MeV. Recent studies have also explored the properties of these materials with various dopants, such as LuAG:Pr, LuAG:Pr,Mo, and LuYAG:Pr.^16–19^ However, all these mentioned materials require advanced synthetic processes of high cost, often involving precise temperature controls and atmospheres, thus remaining a challenge in this research area.

In addition to fast decay times, persistent luminescence behavior under X-ray irradiation has been explored extensively in the quest for more efficient materials for optical information storage, safety signage, anticounterfeiting, theranostics and dosimetry.^20–24^ Persistent luminescence is an optical phenomenon in which the material continues to emit light after the excitation source is removed. This phenomenon is particularly notable in Lu_2_O_2_S:Eu^3+^, which displays long-lasting red emission due to the trapping and delayed release of charge carriers. The addition of codopants such as Mg^2+^ further enhances the duration of afterglow by creating charge compensation defects that facilitate energy storage in trap states. Similarly, Lu_2_O_2_S:Ti^3+^^/^^4+^ exhibits a broad orange emission, attributed to electronic transitions in titanium ions, making it a versatile phosphor for various applications.^25^

The study of scintillators and X-ray-induced persistent luminescent materials relies primarily on analyzing the intensity of the luminescence signal as the excitation energy is scanned across a core absorption edge. X-ray excited optical luminescence (XEOL) refers to the optical emission that occurs following core-level X-ray excitation and can serve as an alternative detection mode for X-ray absorption near edge structure (XANES) analysis. XEOL detection techniques offer additional capabilities beyond conventional XANES spectroscopy, potentially providing information that cannot be accessed through other experimental methods. For instance, XEOL enables site-specific analysis by providing information about the local structure of atoms directly involved in the luminescence emission, offering a significant advantage for XANES investigations.^26–28^

Here, microwave-assisted solid-state synthesis was employed for the first time as a low-cost and alternative method to obtain lutetium-based X-ray phosphors, Lu_2_O_2_S:Eu^3+^ and Lu_2_O_2_S:Ti^3+^^/^^4+^. The synthesis of lutetium oxysulfide is challenging due to the necessity of Lu–S bond formation. According to Pearson’s hard and soft acid–base (HSAB) theory, S^2–^ is a soft base, while lutetium exhibits a harder acid character compared to Y, La, and Gd. Consequently, achieving a high yield requires higher temperatures and an increased sulfur excess. Additionally, codoping effects on their crystal structure and on their optical properties were explored in Lu_2_O_2_S:Eu^3+^,Ti^3+^^/^^4+^, Lu_2_O_2_S:Eu^3+^,Mg^2+^, Lu_2_O_2_S:Ti^3+^^/^^4+^,Mg^2+^, and Lu_2_O_2_S:Eu^3+^,Ti^3+^^/^^4+^,Mg^2+^ materials. XANES and XEOL experiments were carried out to investigate electronic processes excited by X-ray radiation, regarding the contribution of the host and of the dopants to the resulting luminescence mechanism. This work, thus, provides a new understanding for advancing research on lutetium-based scintillators and X-ray-induced persistent luminescence in rare-earth oxysulfide hosts.

## 2 Experimental Section

### 2.1 Synthesis by Microwave-Assisted Solid-State Method

Polycrystalline Lu_2_O_2_S materials were synthesized following an adaptation of a previously established method.^4^ On an agate mortar, stoichiometric amounts of the starting compounds—Lu_2_O_3_ (Xiguanya, 99.99%), Eu_2_O_3_ (Xiguanya, 99.99%), TiO_2_ (Merck, 99.5%), MgCO_3_ (Merck, 99.9%) and S (Sigma-Aldrich, 99.5%) — were mixed and ground with Na_2_CO_3_ (Vetec, 99.5%) used as a flux. The Lu_2_O_3_:S:Na_2_CO_3_ mole ratio was 1:4:0.25. The concentration of dopants, in %-mole with relation to Lu^3+^ site, is described in Table 1.

**Table 1:** Nominal Doping of Lu_2_O_2_S Materials

	% Eu^3+^	% Ti^3+/4+^	% Mg^2+^
Lu_2_O_2_S			
Lu_2_O_2_S:Eu^3+^	5.0		
Lu_2_O_2_S:Ti		1.5	
Lu_2_O_2_S:Mg^2+^			4.5
Lu_2_O_2_S:Eu^3+^,Mg^2+^	5.0		4.5
Lu_2_O_2_S:Ti,Mg^2+^		1.5	4.5
Lu_2_O_2_S:Eu^3+^,Ti	5.0	1.5	
Lu_2_O_2_S:Eu^3+^,Ti,Mg^2+^	5.0	1.5	4.5

For the MASS synthesis, 15 g of granular activated carbon (Ø: 1–2 mm, Synth) was used as a microwave susceptor and placed in a 50 cm^3^ alumina crucible. A second, smaller 5 cm^3^ alumina crucible containing 0.5 g of the precursor powder mixture was positioned inside the larger crucible, surrounded by the susceptor. Both crucibles were partially covered with an alumina lid and placed in a cavity made of aluminosilicate thermal insulation bricks. The precursor powder underwent microwave irradiation in a domestic microwave oven (Electrolux MEF41, 2.45 GHz), using a program set to 10 min at 100% power followed by 15 min at 90% power. The resulting material was ground with 0.6 g of sulfur and the heating procedure was repeated under the same microwave conditions.^4^ The main modifications to the method for Lu_2_O_2_S, in comparison to the oxysulfides Y_2_O_2_S, La_2_O_2_S, and Gd_2_O_2_S, involved the use of higher power and an increased amount of sulfur, both in the precursor mixture (1 Lu_2_O_3_: 4 S ratio) and during the intermediate heating stages.

### 2.2 Characterization

The crystal structures of the materials were evaluated by powder X-ray diffraction (XRD) measurements, using a Bruker D8 DISCOVER diffractometer with Cu Kα radiation (1.5406 Å), in the 2θ range 20–80°, with step size of 0.02° and integration time of 2 s. Rietveld refinements were performed with GSAS II software.^29^ Infrared absorption spectra of were acquired using a PerkinElmer Frontier FTIR spectrometer, in the range from 4000 to 400 cm^–1^, with resolution of 4.0 cm^–1^ and 64 scans. The spectrometer was equipped with an ATR module with a ZnSe sample holder.

Diffuse reflectance spectroscopy (DRS) measurements were performed using a Shimadzu UV 2600 instrument equipped with an integrating sphere, in the range from 200 to 1300 nm, with spectral resolution of 0.1 nm, slit width of 5.0 nm, and integration time of 2 s. X-ray excited optical luminescence (XEOL) and X-ray absorption near edge structure (XANES) measurements at Eu and Lu L_3_-edges were carried out at the Tarumã endstation of Carnaúba beamline of the Brazilian Synchrotron Light Laboratory (Sirius-LNLS),^30^ at the Brazilian Center for Research in Energy and Materials (CNPEM). The samples were positioned with carbon tape on aluminum frames (12 mm 12 mm) with a hole in the center (6 mm diameter). XEOL spectra were acquired at ambient pressure in a special dark chamber to avoid contamination of the signal by external light, using a system composed of an objective lens coated with aluminum (LMM-UVV-15×, Thorlabs), a connecting optical fiber, and a QE Pro UV–vis spectrometer (both from Ocean Insight). XANES spectra at Ti L_2,3_ and Eu M_4,5_-edges were recorded at the XPS endstation of Ipê beamline of Sirius-LNLS.^31^ The treatment of all the synchrotron-based data was performed using PyMCA^32^ and Athena.^33^

Photoluminescence (PL) data were recorded at room temperature (20 °C), using a Horiba Fluorolog-3 FL3-22 instrument fitted with a 450 W xenon lamp excitation source, a single-grating excitation monochromator and a single-grating emission monochromator.

EPR measurements were perform utilizing a CW-Bruker instrument, mod. EMX, operating at X-band (9.5 GHz, 20 mW power, 100 kHz frequency and amplitude modulation 12G), using Wilmad 4 mm quartz tubes, and DPPH (*a*,*a*′-diphenyl-*b*-picrylhydrazyl) as the frequency calibrant (*g* = 2.0036).

## 3 Results and Discussion

### 3.1 Crystal Structure and Phase Purity

To investigate the effect of the matrix and each dopant individually, lutetium oxysulfides doped with Eu, Ti, and Mg (and their possible combinations) were synthesized. The crystal structure and phase purity were examined using X-ray diffraction, with diffraction patterns recorded in the 2θ range of 20° to 80°. Rietveld refinements for all Lu_2_O_2_S XRD patterns converged properly (Figure 1 exemplifies these results for Lu_2_O_2_S:Eu^3+^. For the other samples, see Figures S1–S7). Since the doping levels were relatively high (5% Eu^3+^, 1.5% Ti^3+^^/^^4+^, and 4.5% Mg^2+^), an increase in unit cell volume and lattice parameters was observed, particularly in materials doped with all three ions (i.e., a total of 11% doping) compared to the synthesized pure matrix.

**Figure 1:**
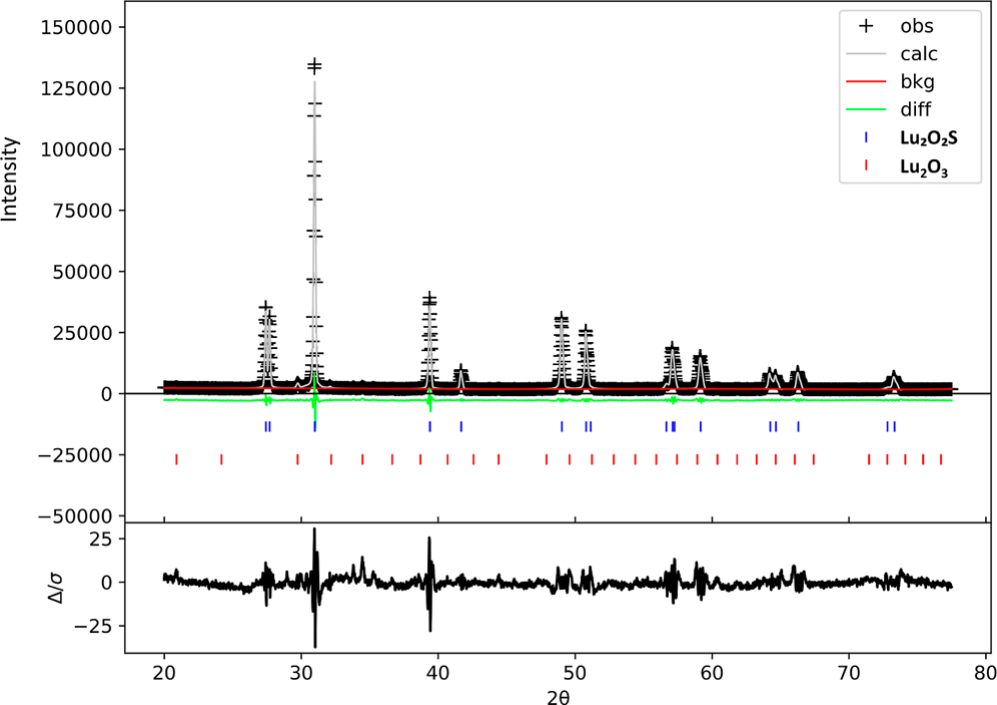
XRD patterns and Rietveld refinement for Lu_2_O_2_S:Eu^3+^ material.

However, no additional phases beyond the target Lu_2_O_2_S host were detected, indicating efficient incorporation of Eu, Ti, and Mg into all materials. Impurities related to the Lu_2_O_3_ precursor (*Ia*3̅ space group) were identified through refinement, with a maximum mass fraction of 6% in all synthesized materials. Obtained values for *R*_p_, *R*_wp_ and χ^2^ parameters can be seen in Tables 2 and 3. Lu_2_O_2_S crystallizes in the trigonal space group (*P*3̅*m*1), in which Lu^3+^ ions are located in sites of *C*_3*v*_ symmetry, surrounded by four oxygen and three sulfur ligands.

**Table 2:** Rietveld Refinement Results for Single-Doped Lutetium Oxysulfide Materials

	Lu_2_O_2_S	Lu_2_O_2_S:Eu^3+^	Lu_2_O_2_S:Ti^3+/4+^	Lu_2_O_2_S:Mg^2+^
Lu_2_O_2_S fraction (% wt)	99.1	96.8	99.9	99.5
nominal doping		5%	1.5%	4.5%
lattice constant (*a* = *b*) (Å)	3.70877	3.70916	3.70898	3.70880
lattice constant (c) (Å)	6.48767	6.48771	6.48770	6.48768
cell volume (Å^3^)	77.282	77.299	77.287	77.285
Lu_2_O_3_ fraction (% w.)	0.9	3.2	0.1	0.5
*R*_p_ (%)	7.40	7.26	5.89	8.39
*R*_wp_ (%)	5.68	5.53	5.78	6.75
χ^2^	6.47	6.35	8.23	6.35

**Table 3:** Rietveld Refinement Results for Co-doped Lutetium Oxysulfide Materials

	Lu_2_O_2_S: Eu^3+^,Ti^3+/4+^	Lu_2_O_2_S:Eu^3+^,Mg^2+^	Lu_2_O_2_S:Ti^3+/4+^,Mg^2+^	Lu_2_O_2_S: Eu^3+^,Ti^3+/4+^,Mg^2+^
Lu_2_O_2_S fraction (% wt)	94.2	98.0	96.6	98.6
nominal doping	5%, 1.5%	5%, 4.5%	1.5%, 4.5%	5%, 1.5%, 4.5%
lattice constant (a = *b*) (Å)	3.70948	3.70938	3.70885	3.70918
lattice constant (c) (Å)	6.48608	6.48682	6.48769	6.48676
cell volume (Å^3^)	77.293	77.290	77.286	77.289
Lu_2_O_3_ fraction (% wt.)	5.8	2.0	3.4	1.4
*R*_p_ (%)	8.16	4.70	5.77	5.34
*R*_wp_ (%)	7.14	4.21	4.45	4.27
χ^2^	8.29	8.08	9.01	9.35

### 3.2 FTIR Measurements

Infrared absorption spectra were recorded for all lutetium oxysulfides and were found to be considerably similar to each other, such that bands exclusively associated with stretching vibrations between S^2–^/O^2–^ and the dopants (Eu^3+^/Ti^3+/4+^/Mg^2+^) were not observed. In the lower-energy region (400–520 cm^–1^), the partially detected absorption bands can be attributed to Lu–S and Lu–O stretching modes, indicating the effective formation of the target matrix. Figure 2 shows the obtained FTIR spectra of Lu_2_O_2_S, Lu_2_O_2_S:Eu^3+^ and Lu_2_O_2_S:Ti. For the other lutetium oxysulfides, the spectra can be seen in Figures S8–S12.

**Figure 2:**
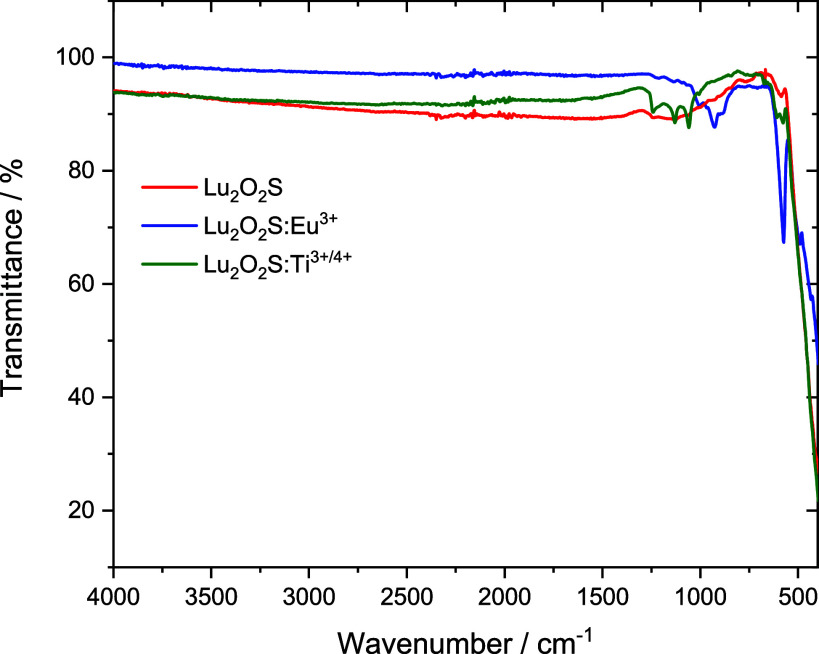
FTIR spectra of Lu_2_O_2_S, Lu_2_O_2_S:Eu^3+^ and Lu_2_O_2_S Ti^3+/4+^ materials.

The absorption bands observed between 1000 and 1200 cm^–1^ are related to the vibrational modes of SO_4_^2–^ ions, which is inconsistent with the structure of oxysulfides, as they do not contain S–O bonds. This finding is similar to previous studies related to other rare earth oxysulfide matrices (La_2_O_2_S, Gd_2_O_2_S and Y_2_O_2_S),^34^ suggesting that the material underwent oxidation after microwave-assisted heat treatment. The investigation of these reduction and oxidation processes, related to synthesis, can be explored through XANES spectral analyses at the sulfur K-edge, which have not yet been performed.

Furthermore, regarding the purity of the materials, no absorption bands characteristic of O–H stretching (∼3500 cm^–1^) or C=O stretching (∼1630 cm^–1^) were observed, indicating the absence of moisture and carbonate, the latter being a potential contaminant due to the use of Na_2_CO_3_ in the synthesis process.^34^

### 3.3 DRS Analysis and Bandgap Determination

Diffuse reflectance spectra (Figure 3 and S13–S16) were recorded to verify the main absorption regions of these materials and identify possible charge transfer transitions. Comparing all materials, a variation in reflectivity is observed in the lower energy region, which can be attributed to the formation of defects during each synthesis, which is an uncontrollable result in MASS synthesis.

**Figure 3:**
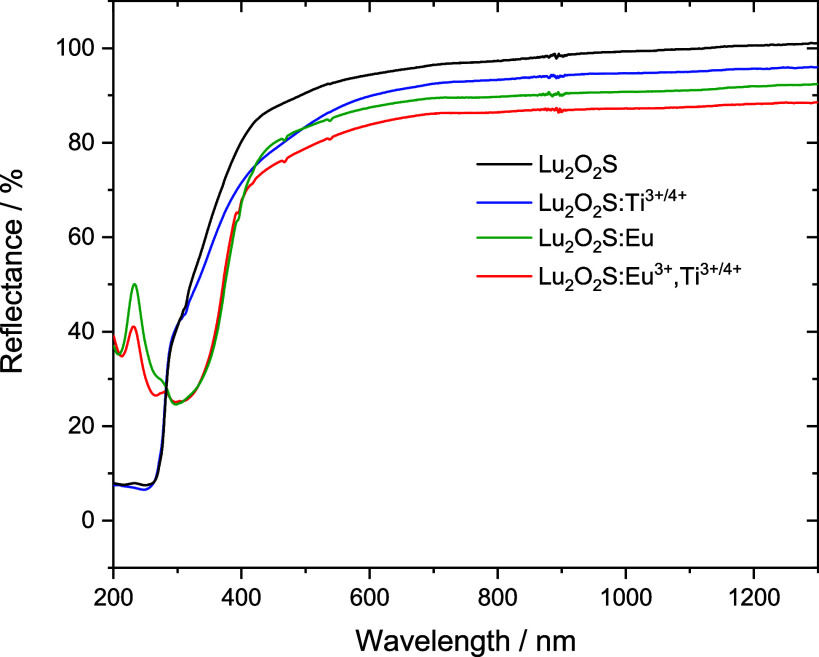
DRS spectra of Lu_2_O_2_S materials.

For undoped Lu_2_O_2_S, an absorption band corresponding to the material’s band gap is observed in the ultraviolet region of the spectrum, whereas for doped materials, the emergence of absorptions in the 200–600 nm region is noticeable, with distinct profiles depending on the dopants or their combination. In the spectra of materials doped with Eu^3+^, a broad absorption band with two maxima can be identified in the UV region, along with 4f–4f transitions of the Eu^3+^ ion superimposed on it. The broad band is related to the ligand-to-metal charge transfer (LMCT) transitions (O^2–^ → Eu^3+^ at 268 nm and S^2–^ → Eu^3+^, at 340 nm) The spectra of Lu_2_O_2_S:Ti^3+/4+^ provided the absorption band energies associated with titanium doping (LMCT O^2–^/S^2–^ → Ti^4+^, at 280 nm).

The interaction between Eu^3+^ and Ti^3+/4+^ is predicted by the persistent luminescence mechanism of these materials and was qualitatively investigated observing the spectra of Lu_2_O_2_S:Eu^3+^, Lu_2_O_2_S:Ti and Lu_2_O_2_S:Eu^3+^,Ti. It is noted that the presence of titanium did not interfere with the occurrence of the LMCT O^2–^/S^2–^ → Eu^3+^, which was observed with high intensity in the codoped matrix. However, the O^2–^/S^2–^ → Ti^4+^ (or prossible Ti^3+^ d–d transitions) do not appear in the spectrum of Lu_2_O_2_S:Eu^3+^,Ti, indicating a possible suppression energy transfer due to the presence of Eu^3+^. Further EPR experiments were conducted aiming to observe possible changes in titanium valence state when comparing Lu_2_O_2_S:Ti and Lu_2_O_2_S:Eu^3+^,Ti materials.

The band gap of the Lu_2_O_2_S host was determined as 4.48 eV using the Kubelka–Munk method (Figure 4), which was higher than the energy observed by Wang et al. (3.94 eV),^35^ but consistent with the value reported by Luo et al. (4.66 eV).^25^

**Figure 4:**
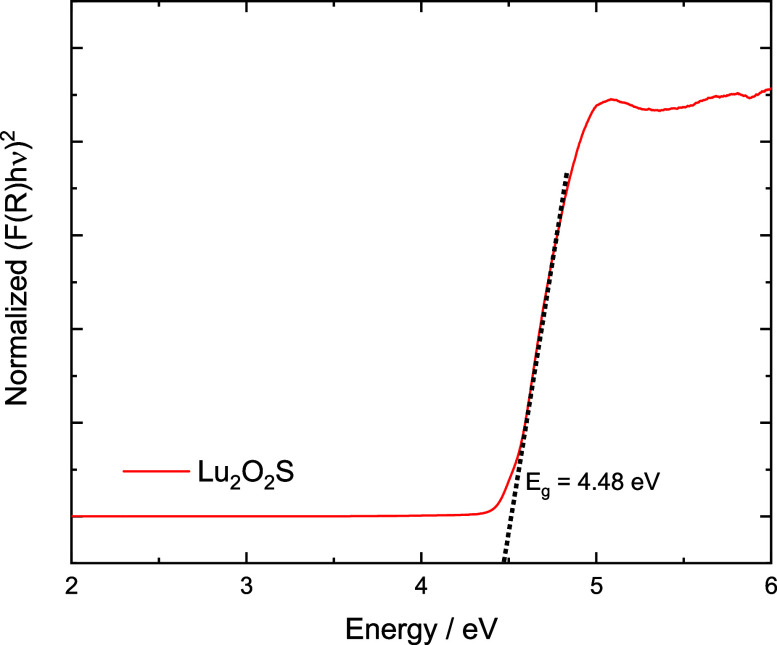
Bandgap calculation for Lu_2_O_2_S host with Kubelka–Munk function.

### 3.4 Luminescent Properties

Figure 5a displays the photoluminescence excitation and emission spectra of doped Lu_2_O_2_S materials. In all cases, the excitation spectra (dashed line) reveal two overlapped absorption bands. The high-energy band, centered at 273 nm, is attributed to the host lattice absorption of Lu_2_O_2_S and aligns with the calculated band gap energy of 4.48 eV. The second band at lower energies, centered around 314 nm, are associated with ligand-to-metal charge transfer (LMCT) transitions: O^2–^(2p) → Eu^3+^(4f^6^) and S^2–^(3p) → Eu^3+^(4f^6^) for Eu-doped materials; and for Ti-doped matrices, O^2–^(2p) → Ti^4+^ and S^2–^(3p) → Ti^4+^ are the most probable processes. It is also noted that the shape of this band varies depending on the dopant combinations. For Eu-doped Lu_2_O_2_S materials, the band profile remains unchanged, even in the presence of Mg^2+^ or Ti. However, Ti bands are present for the materials doped exclusively with Ti or with both Ti and Mg^2+^. This suggests that for Eu^3+^ emission the O^2–^/S^2–^→ Eu^3+^ LMCT transitions are more favorable than those involving O^2–^/S^2–^→ Ti^4+^, exerting stronger influence on the excitation behavior of materials containing both Eu^3+^ and Ti dopants. Additionally, weaker narrow absorption bands corresponding to 4f-4f transitions of the Eu^3+^ ion are observed for Eu-doped matrices, with the most notable being the ^7^F_0_ → ^5^L_6_ transition at 396 nm. It is important to highlight that the excitation spectra of Eu-doped samples were recorded by monitoring the emission at 627 nm, corresponding to the hypersensitive ^5^D_0_ → ^7^F_2_ transition of Eu^3+^. In contrast, for matrices doped with Ti and Ti, Mg^2+^, the excitation spectra were monitored at 605 nm, corresponding to the emission associated with titanium centers.

**Figure 5:**
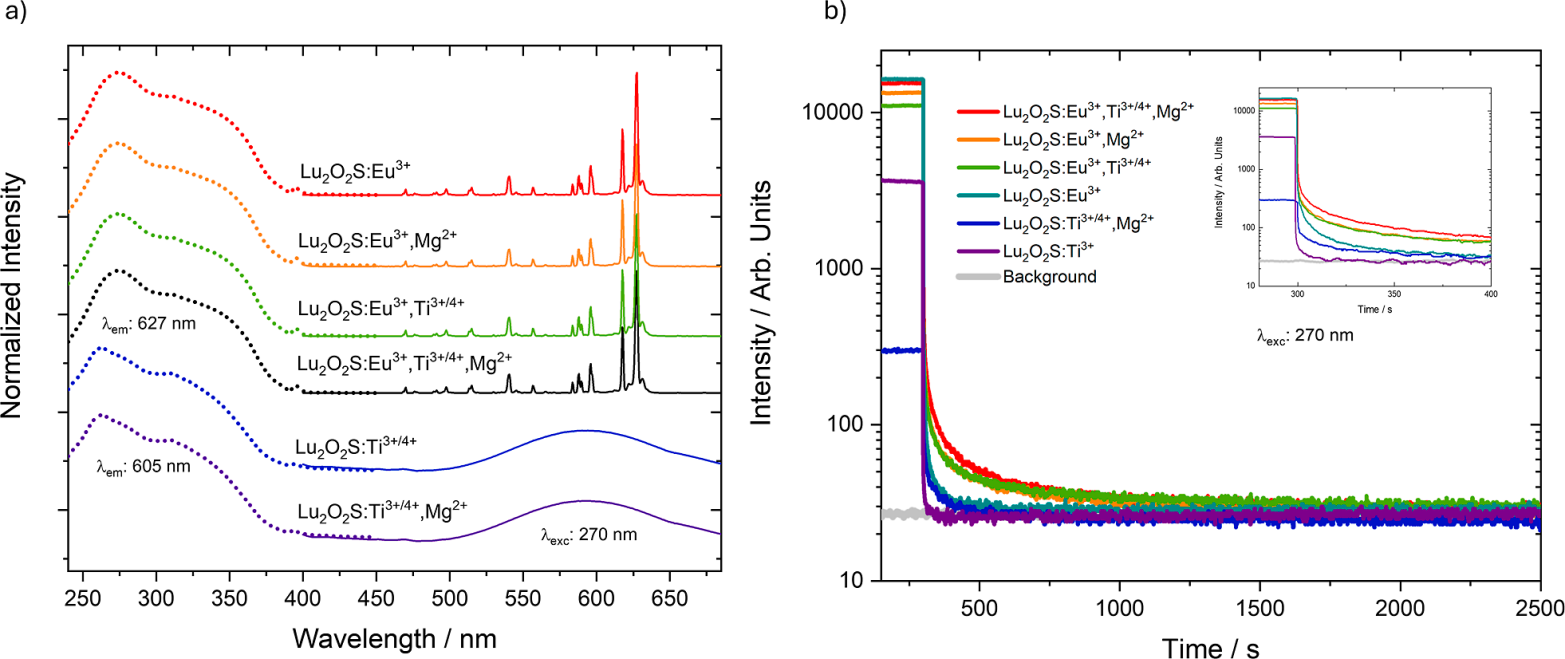
(a) Photoluminescence excitation and emission spectra and (b) persistent luminescence decay curves under 270 nm excitation, monitoring at 605 nm (for Ti^3+/4+^-activated phosphors) and at 627 nm (for Eu^3+^-activated phosphors).

The emission spectra of Eu-doped Lu_2_O_2_S materials, with excitation around the bandgap energy (270 nm) display sharp bands arising from the intraconfigurational 4f-4f transitions of Eu^3+^, with ^5^D_0_ → ^7^F_2_ transition at 627 nm emerging as the most intense emission due to noncentrosymmetric Eu^3+^ sites. As expected, Lu_2_O_2_S:Ti and Lu_2_O_2_S:Ti,Mg^2+^ materials exhibit a broad emission band centered at 605 nm—also under 270 nm irradiation—attributed to radiative recombination processes involving titanium centers, as previously discussed.

Persistent luminescence decay time (Figure 5b) was measured at room temperature, under excitation of 270 nm, for all the samples. As recorded in the excitation spectra, the measurements of Eu-doped samples were performed by monitoring the emission at 627 nm, and for matrices doped with Ti and Ti, Mg^2+^, the persistent luminescence decay curves were monitored at 605 nm. Overall, the luminescence of Lu_2_O_2_S materials activated by Eu^3+^ exhibits a longer persistence compared to those activated by Ti, which indicates that either trapping is more efficient for Eu^3+^ or that nonradiative losses are more efficient on suppressing Ti emission. The introduction of Mg^2+^ increases the persistence duration of the emission of both Lu_2_O_2_S:Eu^3+^ and Lu_2_O_2_S:Ti materials, indicating that the negative defects created by the aliovalent doping are effective in hole-storage for these materials. The presence of Ti in Lu_2_O_2_S:Eu^3+^,Ti also contributes positively to persistent luminescent behavior, not only by introducing defects in the host—and consequently trap levels of adequate depth—but also by being a center in which an energy transfer to Eu^3+^ possibly occurs. The mechanism of persistent luminescence in rare-earth oxysulfides, especially involving the Ti → Eu energy transfer in Eu, Ti-doped materials, has been widely explored in the literature.^34,36–38^ However, the nature of the charge carriers involved in the trapping and detrapping processes remains unclear, as well as the valence state of titanium. Lu_2_O_2_S:Eu^3+^,Ti,Mg^2+^ exhibited the longest-lasting persistent luminescence, as expected, which is a result of a greater number of defects, including charge-compensation and vacancies.

XEOL emission spectra for Lu_2_O_2_S:Eu^3+^, Lu_2_O_2_S:Ti and Lu_2_O_2_S:Eu^3+^,Ti were recorded in the visible region under excitation at the Eu L_3_-edge (edge peak at 6983 eV) and Lu L_3_-edge (edge peak at 9248 eV). For Eu-doped samples (Figure 6a,b), these spectra were consistent with what was observed in the UV-excited spectra, displaying the expected profile for Eu^3+^-doped materials: characteristic 4f–4f transitions, with ^5^D_0_ → ^7^F_2_ transition at 627 nm being the most intense, which is typical for sites without an inversion center. The emission profile remained identical under both excitation energies, indicating that the X-ray excited luminescence in these materials is independent of the incident photon energy. Additionally, no variations in the intensities of the 4f–4f transition peaks were observed, suggesting that the excitation mechanism does not alter the population of the electronic states involved in the emission transitions, regardless of whether Eu or Lu is directly excited. Lu_2_O_2_S:Ti material exhibited a broad emission band (Figure 6c), related to titanium centers, under both X-ray excitation energies, but with a redshift to 631 nm compared to the emission spectra obtained under UV excitation. This result suggests that X-ray irradiation may induce additional electronic processes that affect the distribution of excited states, thereby altering the radiative recombination energy. When a material is exposed to X-ray excitation, high-energy photons interact with the atomic structure, leading to the creation of high-energy electron–hole pairs (excitons) and secondary electrons that further ionize atoms in a cascade effect. This process significantly increases the number of free charge carriers within the material. In persistent luminescent materials, trap levels can capture the free electrons or holes generated by X-rays, temporarily storing them before recombination. This process significantly impacts charge carrier dynamics and alters the resulting luminescent properties.^39^ However, since the valence state of titanium in this host is not well stablished, more experiments should be conducted to elucidate the nature of these titanium emissions.

**Figure 6:**
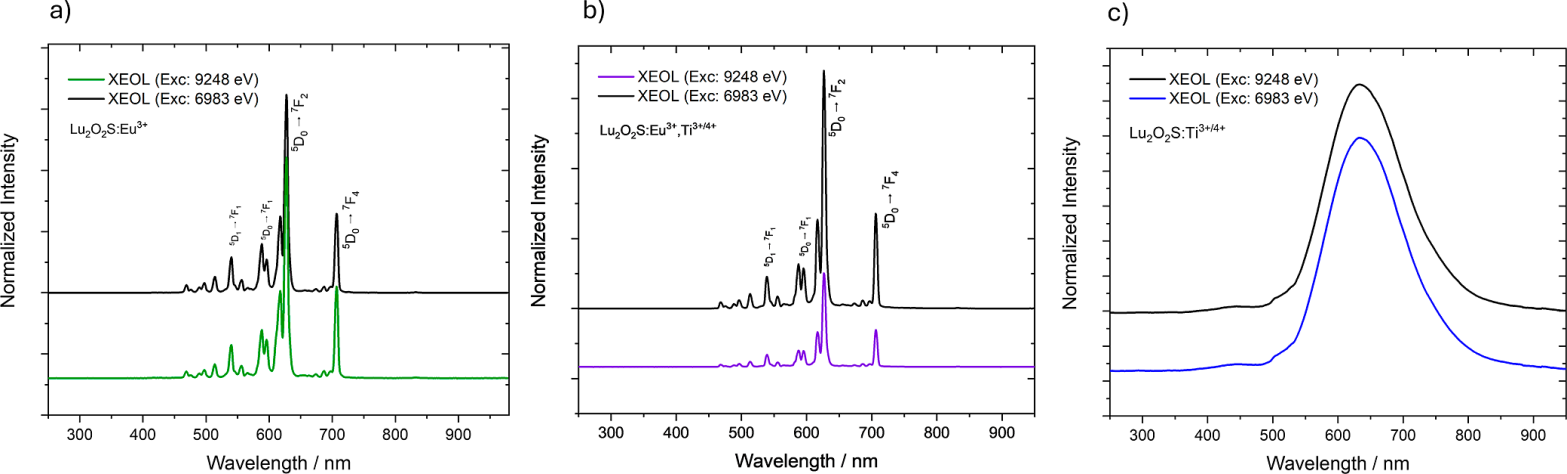
XEOL emission spectra under excitation at Eu and Lu L_3_-edges for (a) Lu_2_O_2_S:Eu^3+^, (b) Lu_2_O_2_S:Eu^3+^,Ti^3+/4+^ and (c) Lu_2_O_2_S:Ti^3+/4+^, recorded at room temperature in the visible region.

### 3.5 Synchrotron Radiation Studies: XANES and XEOL

The valence states of the dopants Eu and Ti in the Lu_2_O_2_S matrices were studied with XANES measurements at the L_2_, L_3_ edges of Ti and the M_4_, M_5_ edges of Eu. The presence of Ti^3+^ or Ti^4+^ in these materials, particularly in the single-doped matrices where emission originates from titanium doping, has not yet been elucidated in the literature. The titanium species associated with the observed emission in the orange region also remains a subject of study in the area, not only for Ti-doped Lu_2_O_2_S, but also for Y_2_O_2_S, La_2_O_2_S and Gd_2_O_2_S.

It is proposed that if the emitting species is Ti^3+^, the observed emission originates from a d–d transition. However, the same emission could also arise from LMCT (O^2–^/S^2–^ → Ti^4+^) states if the activator is the tetravalent species. The spectra at the L_2_, L_3_ edges of Ti were recorded for Lu_2_O_2_S:Ti and Lu_2_O_2_S:Eu,Ti, as well as for Ti_2_O_3_ and TiO_2_, used as reference materials for the trivalent and tetravalent species, respectively. It is noted that the profiles of Ti_2_O_3_ and TiO_2_ are relatively similar in both shape and energy, making the analysis challenging.

By examining the sample profiles (Figure 7a), the presence of Ti^3+^ and Ti^4+^ can be inferred in all materials. However, these results remain not conclusive. As observed in some codoped persistent luminescent materials,^39,40^ it is considered that during measurement, part of the emitting center can be temporarily oxidized/reduced, making XANES insufficient to determine the exact concentration of each species. Complementary analyses, such as XPS, would therefore be of great interest for this study.

**Figure 7:**
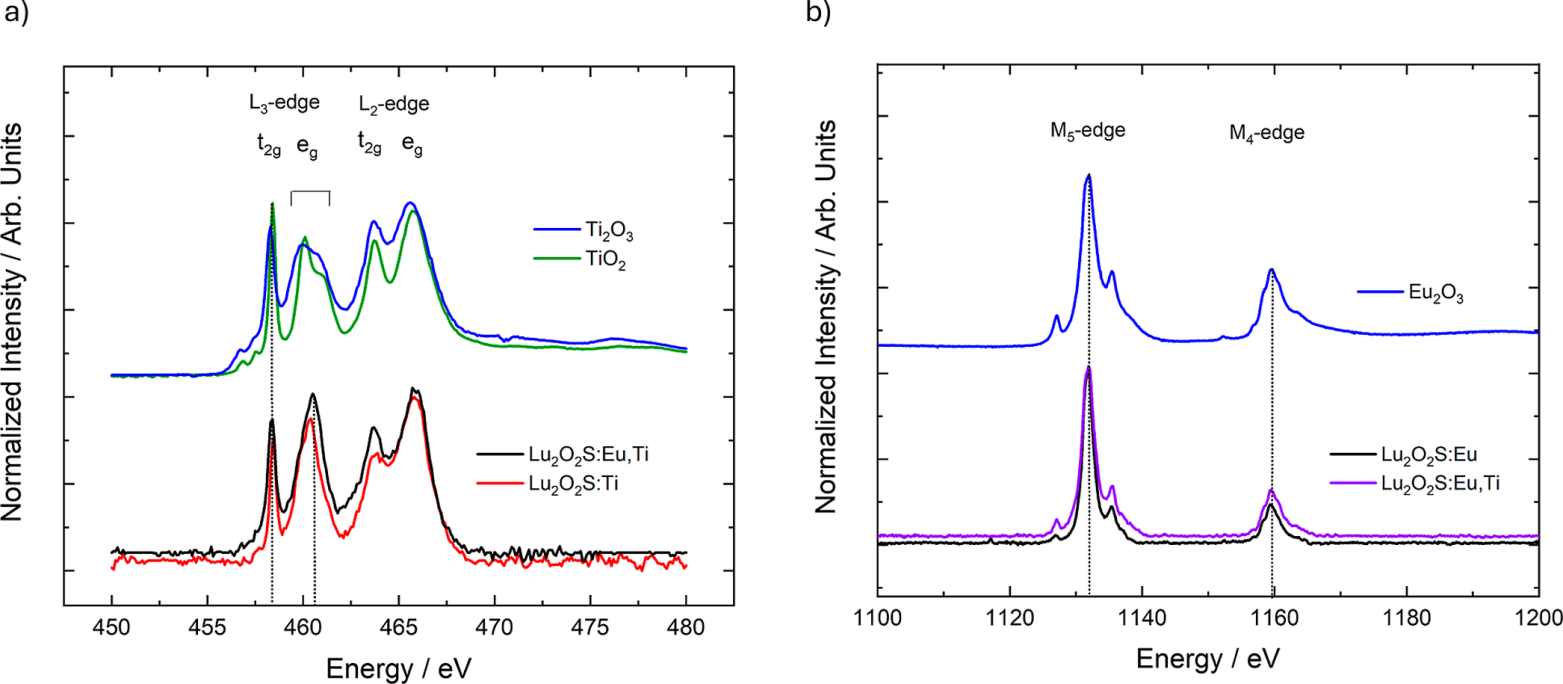
XANES spectra of Lu_2_O_2_S:Eu^3+^, Lu_2_O_2_S:Eu^3+^,Ti^3+/4+^ and Lu_2_O_2_S:Ti^3+/4+^ at (a) Ti L_2_,L_3_-edge and (b) Eu M_4_,M_5_-edge. TiO_2_, Ti_2_O_3_ and Eu_2_O_3_ were measured as standards for Ti^4+^, Ti^3+^ and Eu^3+^ species, respectively.

The spectra at the M_4_, M_5_ edges of Eu were recorded for Eu_2_O_3_, Lu_2_O_2_S:Ti, and Lu_2_O_2_S:Eu,Ti (Figure 7b). The sample profiles were similar to the reference material’s profile, strongly indicating the presence of Eu^3+^ in all synthesized materials, regardless of the presence of the codopant.

XANES and total XEOL yield were also recorded around Eu L_3_-edge and Lu L_3_-edge (Figures 8–10). Both the XANES and XEOL excitation spectra were normalized to the initial intensity of the radiation beam (*I*_0_) and measured simultaneously.

**Figure 8:**
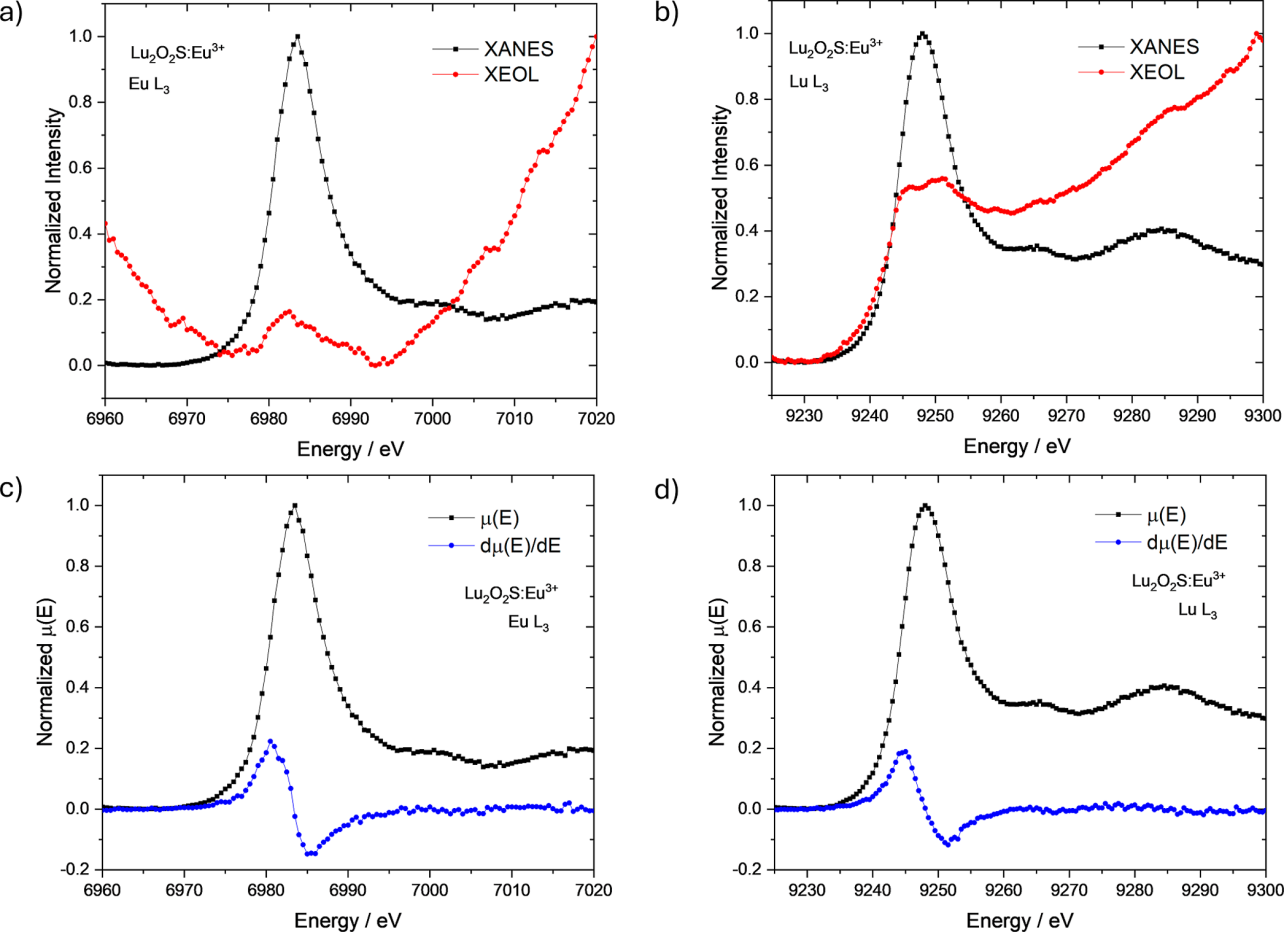
XEOL and XANES spectra of Lu_2_O_2_S:Eu^3+^ at (a) Eu L_3_-edge and (b) Lu L_3_-edge. XANES spectra and the respective first derivatives at (c) Eu L_3_-edge and (d) Lu L_3_-edge.

In Figure 8, Lu_2_O_2_S:Eu^3+^ material was measured in the X-ray energies around the Eu L_3_-edge (Figure 8a) and Lu L_3_-edge (Figure 8b). It is observed that the region of maximum X-ray absorption by the Eu atom corresponds to an overall decrease in optical luminescence intensity, with a subtle increase at the edge peak, indicating a positive XEOL response to the resonance energy (2p4f^6^ → 2p4f^6^5d* transition). However, the prevailing decrease in luminescence signal may be attributed to competing processes that occur after X-ray absorption in this energy range, such as fluorescence or Auger electron emission, especially at the pre-edge region (2p4f^6^ → 2p4f^6^4f* or 2p4f^6^ → 2p4f^6^(4f/5d)* transitions, in which 4f/5d denotes an hybrid final state). The XEOL at the Lu L_3_-edge, however, exhibited a positive edge in this energy range, suggesting that direct excitation of the Lu atom in the host matrix promotes the material’s emission and is therefore linked to the luminescence mechanism. The luminescence intensity profile also follows exactly the XANES pre-edge shape, which indicates that both 2p4f^14^ → 2p4f^14^5d* (edge) and 2p4f^14^ → 2p4f^14^(4f/5d)* (pre-edge) transitions contribute to the mechanism. In this scenario, optical luminescence may occur through the following processes: (i) X-ray absorption by the Lu atom in the matrix, leading to the formation of an electron–hole pair; (ii) delocalization and transport of the excited electron along the conduction band; and (iii) radiative recombination in the optical channel of Eu.

XANES spectrum and the respective first derivative at the Eu L_3_-edge (Figure 8c) indicates the predominant presence of the trivalent Eu^3+^ species compared to the divalent Eu^2+^, associated with the peaks at 6983 and 6975 eV, respectively, as expected for this material. Despite the reducing atmosphere used during the synthesis process, the results consistently show the presence of Eu^3+^, which could also be favored by factors such as sample oxidation and the temporary oxidation of Eu^2+^ to Eu^3+^ during X-ray experiments. The spectrum at the Lu L_3_-edge (Figure 8d) also confirmed the complete presence of Lu^3+^, as highlighted by the first derivative. Eu-L_3_ and Lu L_3_-edge XANES spectra of all synthesized materials (Lu_2_O_2_S:Eu^3+^, Lu_2_O_2_S:Ti and Lu_2_O_2_S:Eu^3+^,Ti) were found to be identical, indicating the predominant presence of both trivalent species, Eu^3+^ and Lu^3+^ (Figures 8c,d, 9b and 10c,d).

**Figure 9:**
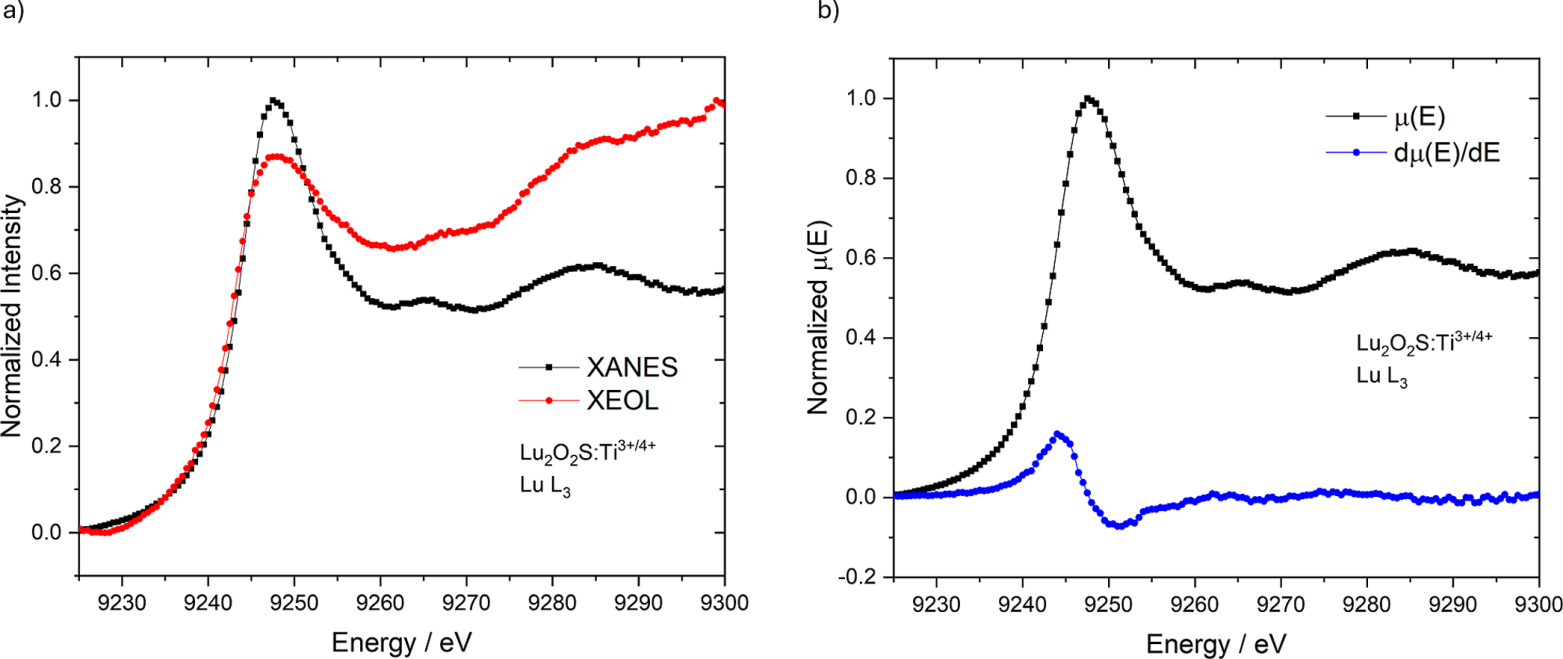
(a) XEOL and XANES spectra of Lu_2_O_2_S:Ti^3+/4+^ at Lu L_3_-edge. (b) XANES spectra and the first derivative at Lu L_3_-edge.

In Figure 9, XEOL and XANES at the Lu L_3_-edge are presented for Lu_2_O_2_S:Ti material. As observed for Lu_2_O_2_S:Eu^3+^, a positive XEOL signal was detected within this energy range, but with greater intensity (Figure 9a). This suggests that the luminescence mechanism is similar; however, the contribution of absorption by the lutetium atom appears to play a more significant role in Lu_2_O_2_S:Ti emission. Consequently, the emission from titanium in this host is more efficiently promoted by X-ray excitation than that of Eu^3+^. This could be attributed to a higher number of competing processes triggered by X-rays in the presence of Eu^3+^, as its higher atomic number (*Z*) makes it a more effective X-ray absorber compared to Ti. A similar analysis was conducted for Lu_2_O_2_S:Eu^3+^,Ti^3+/4+^, where a comparable overall decrease in luminescence intensity was observed at the Eu L_3_-edge; however, no positive XEOL signal was detected at the edge peak (Figure 10a). In contrast, the XEOL at the Lu L_3_-edge displayed a positive edge in this energy range, confirming that direct excitation of the Lu atom contributes significantly to the material’s emission (Figure 10b). These results, furthermore, suggest that X-ray-excited luminescence in this host follows the same mechanism for both Eu^3+^ and Ti activators, where direct excitation of the host lattice is likely the dominant pathway. In comparison, excitation through the Eu channel might involve additional competing processes following the exciton formation for both Eu-doped materials.

**Figure 10:**
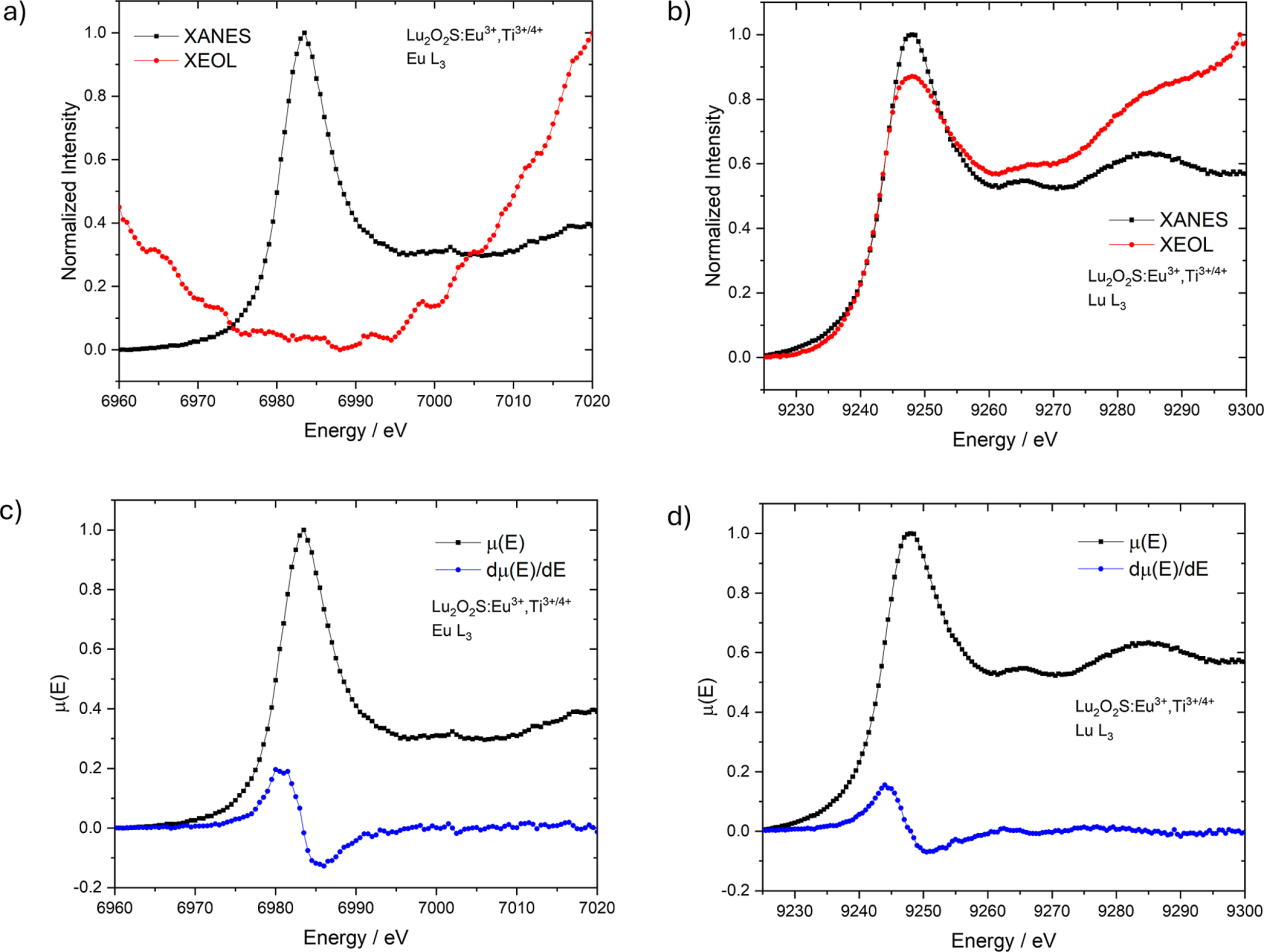
XEOL and XANES spectra of Lu_2_O_2_S:Eu^3+^,Ti^3+/4+^ at (a) Eu L_3_-edge and (b) Lu L_3_-edge. XANES spectra and the respective first derivatives at (c) Eu L_3_-edge and (d) Lu L_3_-edge.

The EPR spectra of Lu_2_O_2_S:Ti and Lu_2_O_2_S:Eu,Ti were recorded both in the absence and presence of UV irradiation (Figures 11 and S17–S18). For the Ti-doped sample, no significant signal was detected, indicating the absence of a measurable amount of Ti^3+^, either in the dark or under UV exposure. This may be attributed to the inherently low intensity of the Ti-EPR signal, which results from the low natural abundance (∼13%) of the EPR-active isotopes ^47^Ti and ^49^Ti.

**Figure 11:**
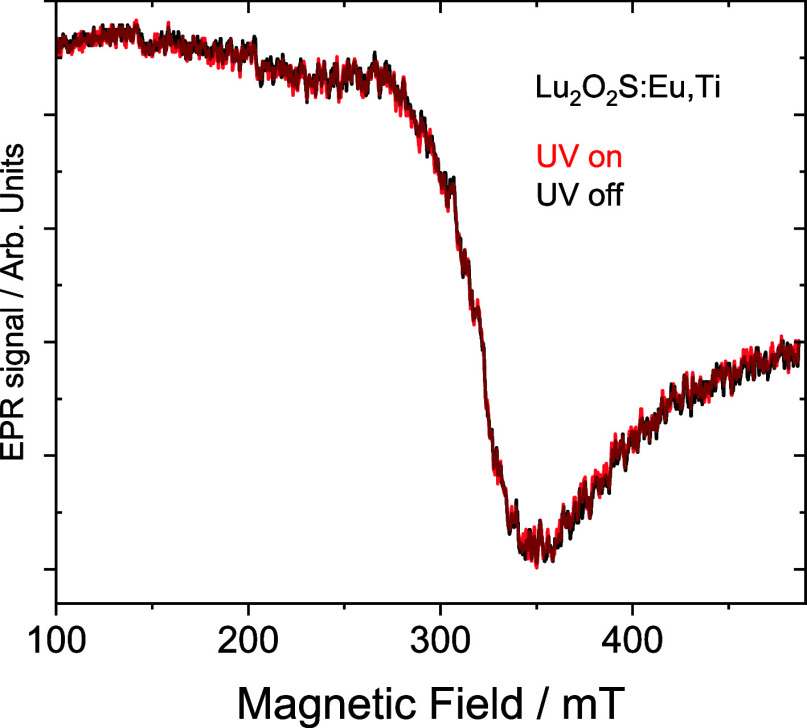
EPR spectra of Lu_2_O_2_S:Eu,Ti with and without UV irradiation.

In contrast, the Eu, Ti-doped sample exhibited a broad signal with *g* = 2.07, which remained unchanged upon UV irradiation. This *g*-value is higher than the typical value for pure Ti^3+^ in oxide materials (∼1.99). However, to date, EPR studies of Ti^3+^ in sulfide hosts are scarce in the literature, making it challenging to precisely attribute the observed EPR signal in the oxysulfide host, particularly given its higher covalency compared to oxides. Notably, increased covalency has been reported to shift g-values, as observed for Mn^2+^ and Fe3+,^42,43^ suggesting that this effect may also influence the EPR parameters in the present system leading to higher g-values of Ti^3+^. On the other hand, a similar signal has been reported in standard TiO_2_ samples,^41^ where it is associated with surface hole-trapping sites in titania photocatalysts. This makes the attribution of the oxidation state in our system to be inconclusive.

Regardless of whether the observed signal originates from Ti^3+^ or Ti-related hole-trapping sites, it is crucial to emphasize that its intensity and shape remain unchanged under UV irradiation. This indicates that the charge-trapping mechanism responsible for the persistent luminescence in these materials does not significantly alter the electronic structure of Ti. Moreover, no Eu^2+^ signal was detected, confirming the absence of photoredox processes in the persistent luminescence mechanism. This behavior contrasts with the mechanism observed in Eu^2+^-based systems, where photoredox activity has been demonstrated.

It is important to note that, despite the efficient induction of persistent luminescence by X-ray radiation, Eu^2+^ is also not observed in any XANES spectra. This finding is consistent with EPR measurements, which indicate the absence of photoredox processes. These results suggest that the charge separation involved in the charge-carrier trapping process for Eu^3+^-based persistent luminescent materials (with or without Ti codoping) is not as pronounced as in Eu^2+^-based materials. Consequently, no formal photoredox processes occur, meaning Eu^2+^ is not detected in XANES.

This hypothesis is supported by the following considerations:

1. In Eu^2+^, the ground state consists of a localized 4f orbital, whereas the excited state is more delocalized due to the mixing of 5d orbitals with the conduction band, resulting in significant charge separation and subsequent photooxidation of Eu^2+^ during the trapping process.
2. In contrast, for Eu^3+^—particularly under ligand-to-metal charge transfer (LMCT) excitation, which is more efficient in generating persistent luminescence—the ground state is primarily composed of oxygen 2p orbitals in close proximity to Eu^3+^, while the excited state consists of localized 4f orbitals.
3. A similar behavior to Eu^2+^ is expected for Ti^3+^, as its excited state (3d) is more delocalized due to mixing with the conduction band. Conversely, Ti^4+^ is expected to resemble Eu^3+^, as its excitation mechanism also involves LMCT.

The implications of this hypothesis are as follows:

(i) The Ti species involved in persistent luminescence are likely Ti^4+^, given that no changes were detected in EPR measurements under UV irradiation, suggesting that no formal charge separation occurs.
(ii) The hole-trapping sites may be located in close proximity to the emitting centers, effectively reducing the spatial separation of charge carriers.

To further validate this hypothesis, theoretical calculations should be conducted to evaluate the density of states of these materials, allowing for a more detailed analysis of the composition, localization of relevant electronic states, and potential defect positions. However, such an investigation falls beyond the scope of this work.

## 4 Conclusion

Eu-, Ti- and Mg-doped Lu_2_O_2_S materials were synthesized with high yield through MASS method for the first time, using 100 times less energy than conventional solid-state synthesis. The observed oxide impurities did not exceed 3.5% in weight for all samples. In XRD experiments, it was observed that the incorporation of dopants induces an increase in unit cell volume and lattice parameters. The FTIR spectra of the oxysulfides, consistent with the XRD analysis, confirmed that the samples were obtained with high purity, as no absorption bands related to water or carbonate were detected. However, the presence of sulfate vibrational modes suggested possible oxidation of the materials after thermal treatment. XANES measurements at the Eu L_3_-edge for all materials indicated a predominance of the trivalent Eu^3+^ and Lu^3+^ species. Together with XEOL spectra, the excitation and emission profiles under different X-ray energies were analyzed. Combining XEOL and XANES spectra at Eu L_3_ and Lu L_3_-edges, the contribution of europium and lutetium absorptions to the optical luminescence intensity was investigated. Although Eu^3+^ acts as an activator, the optical luminescence of the materials was not enhanced by X-ray absorption at the Eu L_3_-edge. In contrast, absorption by the matrix at the Lu L_3_-edge indeed induced luminescence and positively contributed to the mechanism. The XEOL emission spectra remains identical under different X-ray excitation energies for each material. In the case of Eu,Ti-doped Lu_2_O_2_S, only Eu^3+^ transitions were observed under both UV and X-ray irradiation, which indicates the occurrence of a Ti → Eu energy transfer process, in agreement with the results obtained with UV-spectroscopy. Under UV irradiation, codoped materials show longer persistent luminescence decay times in comparison with single-doped matrices, as a consequence of a greater number of defects. Eu^3+^-activated Lu_2_O_2_S materials exhibit greater persistence in emission compared to those activated by Ti, due to the nature of defects and the trap depth introduced by doping. EPR and XANES analyses provide strong evidence that the persistent luminescence mechanism in Lu_2_O_2_S:Eu,Ti does not involve a formal photoredox processes. EPR showed to be inconclusive on Ti-oxidation state due to the few data available on covalent Ti^3+^ systems. Nevertheless, the absence of changes under irradiation indicates that Ti-oxidation state remained unaffected by UV. Additionally, the absence of an Eu^2+^ signal in both XANES and EPR spectra suggested that the charge separation that occurs in Eu^3+^-based persistent luminescent materials is not as pronounced as in Eu^2+^-based systems, preventing formal photoredox processes. These findings highlight key differences in the luminescence mechanisms of Eu^3+^- and Eu^2+^-doped materials, providing valuable insights into their electronic and structural properties. Eu- and Ti-doped lutetium oxysulfides demonstrated promising potential for radioluminescence applications, effectively converting X-ray irradiation into visible light across different excitation energies.

## Supplementary Material



## Data Availability

The authors declare that all data underlying the results are available as part of the main article and the data supporting this article have been included as part of the Supporting Information. No additional source data are required.

## Notes

X-ray diffractograms with Rietveld refinement and infrared spectra of pure and Eu-, Ti-, Mg- doped Lu_2_O_2_S materials; EPR spectra of Lu_2_O_2_S:Ti and Lu_2_O_2_S:Eu,Ti with and without UV irradiation; Experimental setup of microwave-assisted solid-state synthesis (PDF)
