# Supplementary material for: XEOL and Persistent Luminescence in Eu- and Ti-Doped Lu2O2S Materials
Source: ACS Omega. 2025 Jul 22;10(30):33317–27. doi: 10.1021/acsomega.5c03406 (PMC12332611; doi:10.1021/acsomega.5c03406)
Supplement: Supplementary file 1 [file ao5c03406_si_001.pdf]

XEOL and Persistent Luminescence in Eu- and Ti-doped Lu_2O_2S materials

Karina T. Fonseca[a], Nataly S. Santos[a], Marcelo C. Portes[a], Fernando A. Garcia[b] and Lucas C. V. Rodrigues[a,*]

[a] Department of Fundamental Chemistry, Institute of Chemistry, University of São Paulo, São Paulo-SP, 05508-000, Brazil

[b] Department of Applied Physics, Institute of Physics, University of São Paulo, São Paulo-SP, 05508-900, Brazil

*Corresponding author: Lucas Carvalho Veloso Rodrigues

Departamento de Química Fundamental, Instituto de Química, Universidade de São Paulo, São Paulo, SP, Brazil.

Phone:

E-mail: lucascvr@iq.usp.br

SUPPLEMENTARY MATERIAL

1. *XRD patterns with Rietveld refinement*

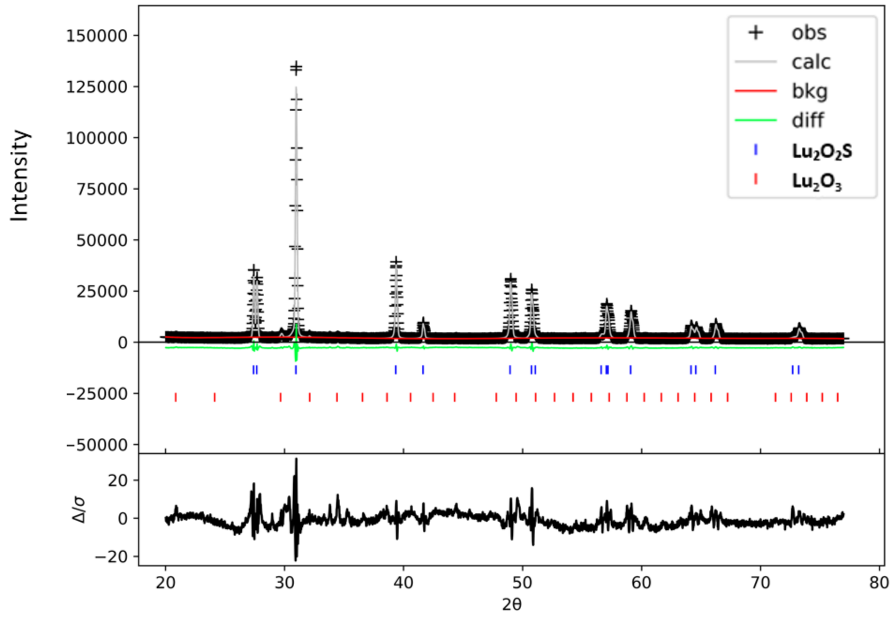

Figure S1. XRD data of $Lu_2O_2S:Eu^{3+},Mg^{2+}$ material.

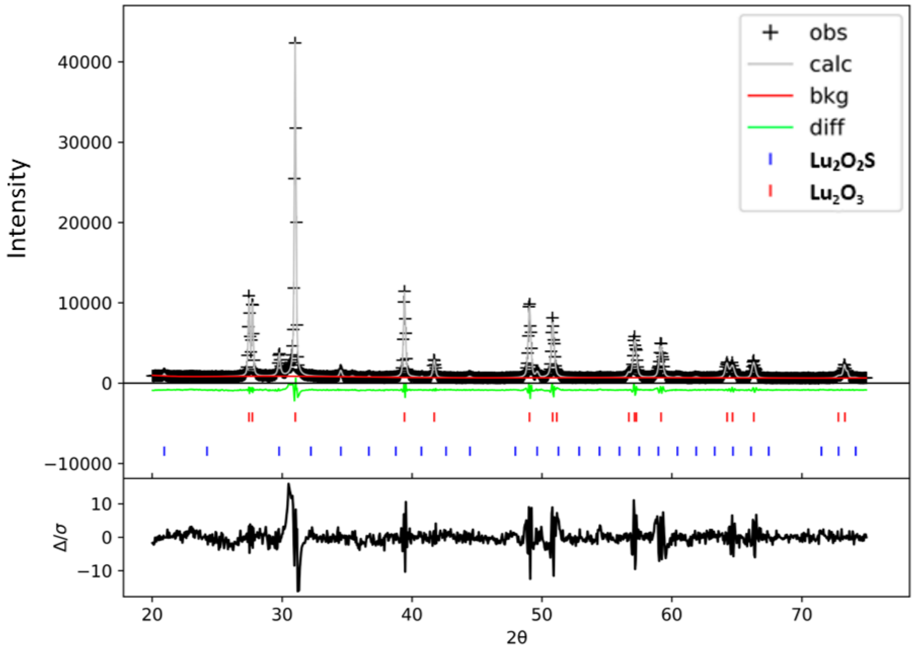

Figure S2. XRD data of $Lu_2O_2S:Eu^{3+},Ti^{3+/4+}$ material.

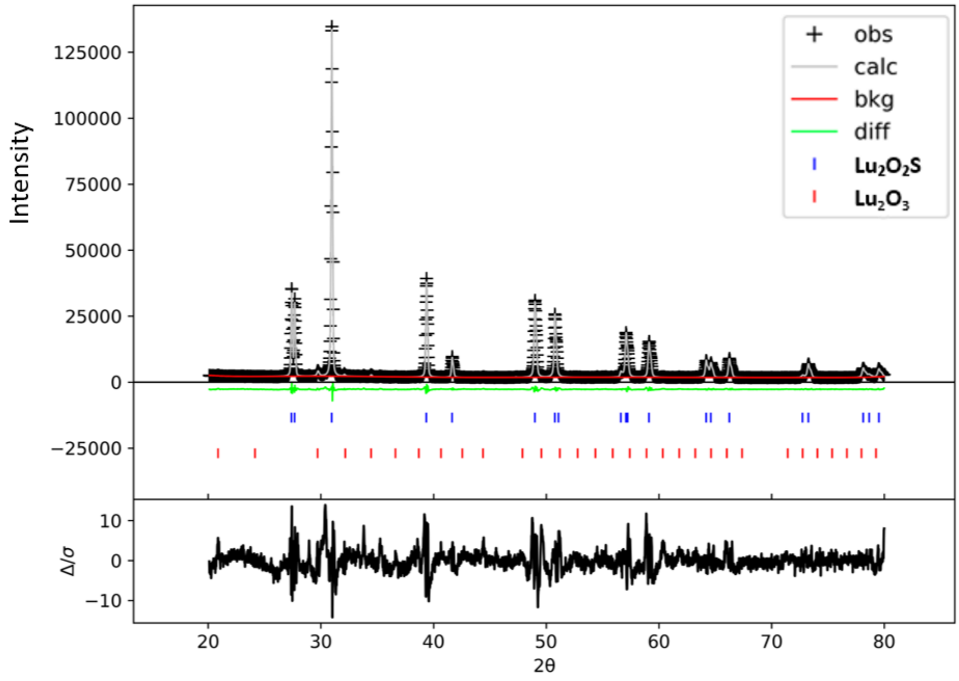

Figure S3. XRD data of $Lu_2O_2S:Eu^{3+},Ti^{3+/4+},Mg^{2+}$ material.

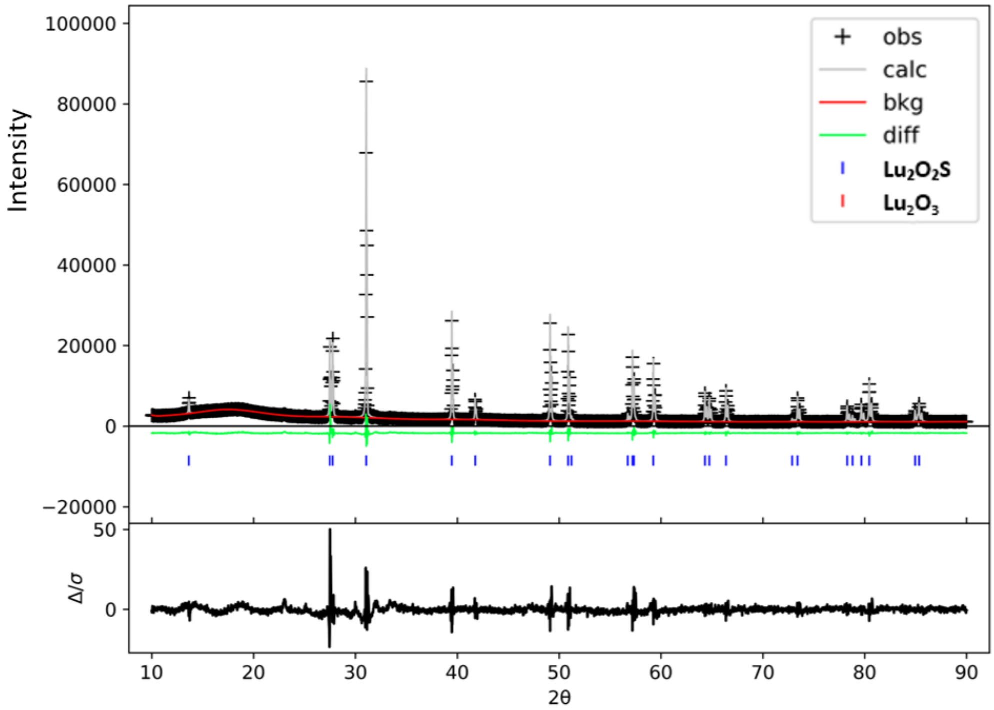

Figure S4. XRD data of $Lu_2O_2S:Mg^{2+}$ material.

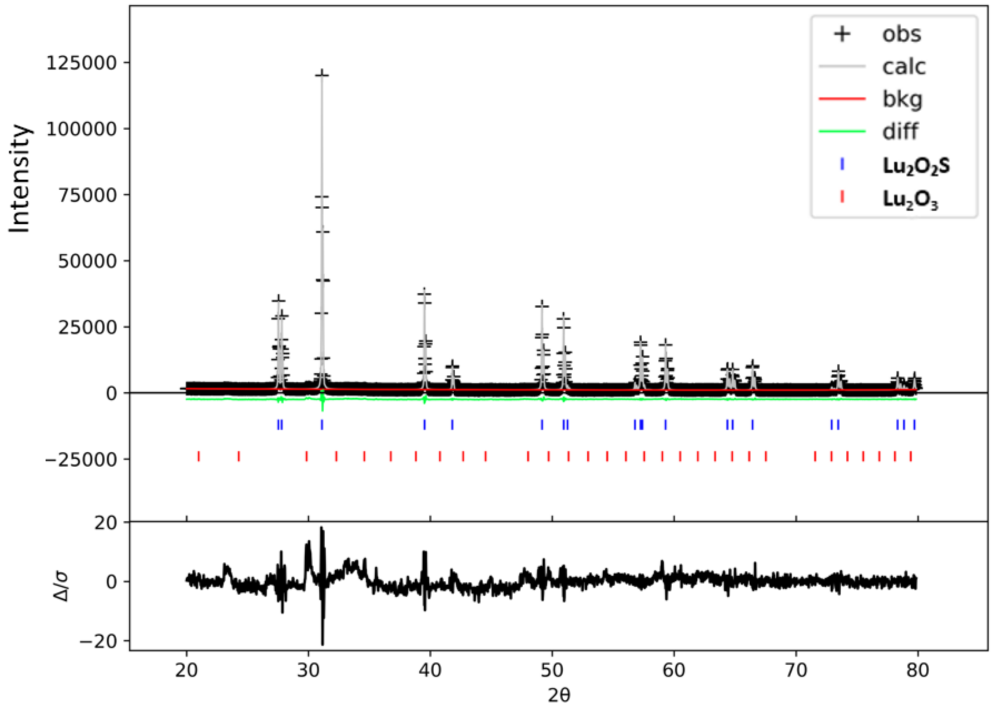

Figure S5. XRD data of Lu_2O_2S material.

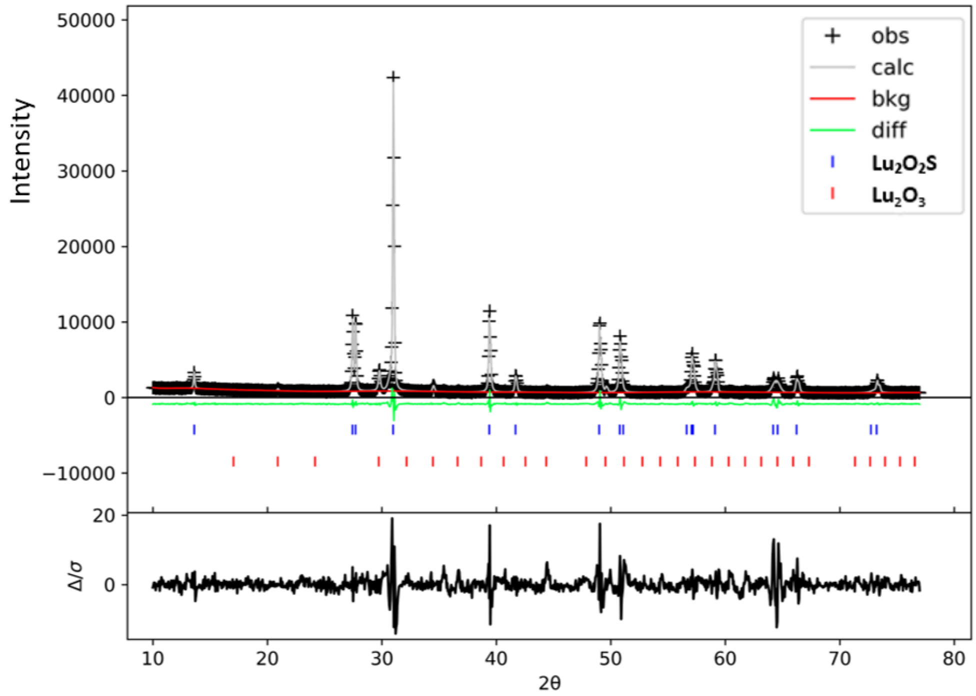

Figure S6. XRD data of $Lu_2O_2S:Ti^{3+/4+}$ material.

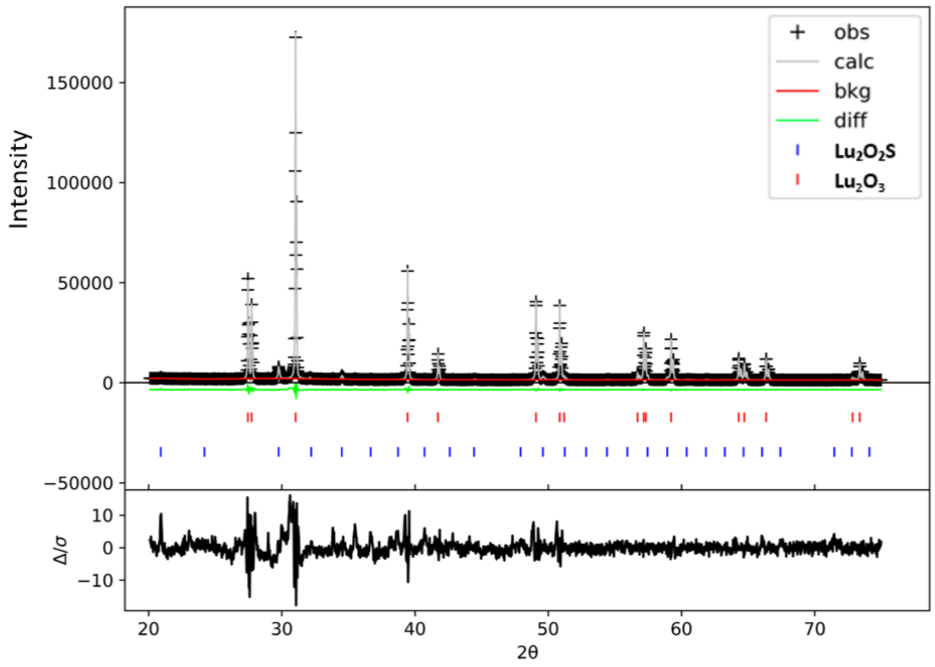

Figure S7. XRD data of $Lu_2O_2S:Ti^{3+/4+},Mg^{2+}$ material.

REFERENCES

Crystallographic Information Files

Lu_2O_2S

Drafall, L. E., McCarthy, G. J., Sipe, C. A. & White, W. B. ON THE PREPARATION AND X-RAY POWDER DATA OF RARE EARTH SULFIDES AND OXYSULFIDES. 954–961 (1974).

Lu_2O_3

Guzik, M. *et al.* Structural investigations of Lu2O3 as single crystal and polycrystalline transparent ceramic. *Crystal Growth and Design* **14,** 3327–3334 (2014).

2. *FTIR spectra*

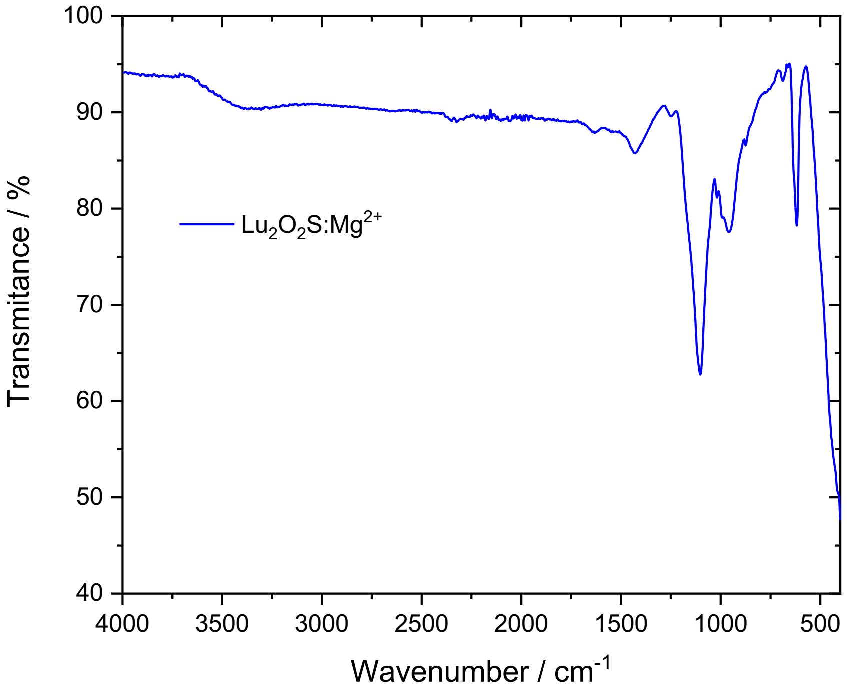

Figure S8. FTIR spectra of $Lu_2O_2S:Mg^{2+}$ material.

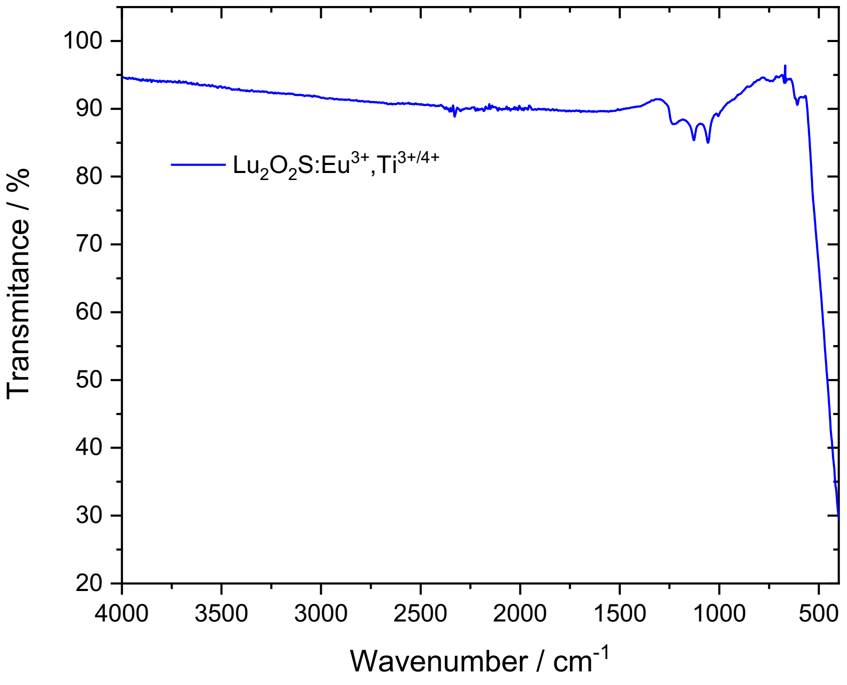

Figure S9. FTIR spectra of $Lu_2O_2S:Eu^{3+},Ti^{3+/4+}$ material.

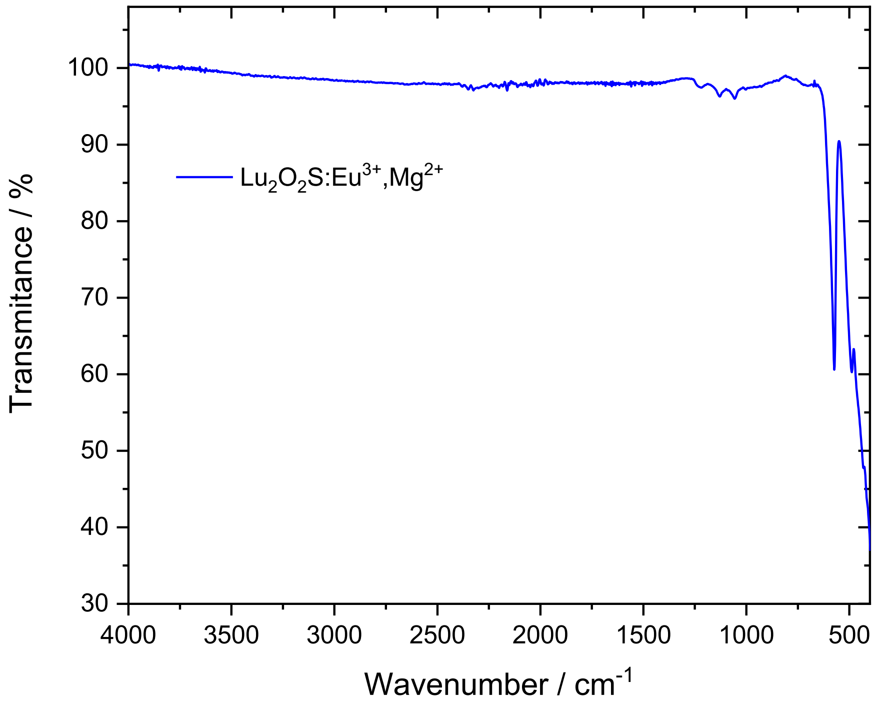

Figure S10. FTIR spectra of $Lu_2O_2S:Eu^{3+},Mg^{2+}$ material.

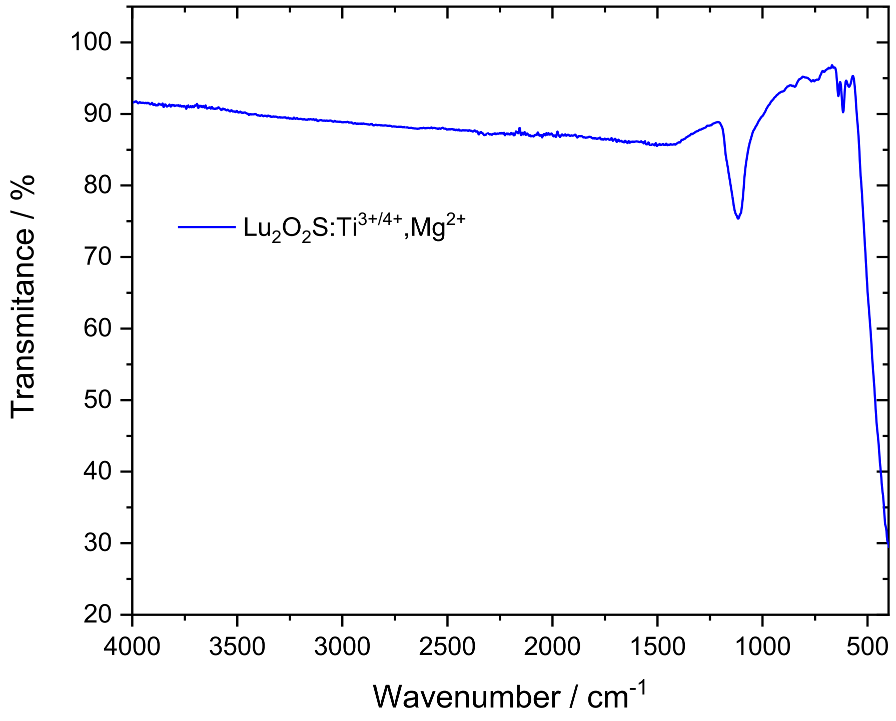

Figure S11. FTIR spectra of $Lu_2O_2S:Ti^{3+/4+},Mg^{2+}$ material.

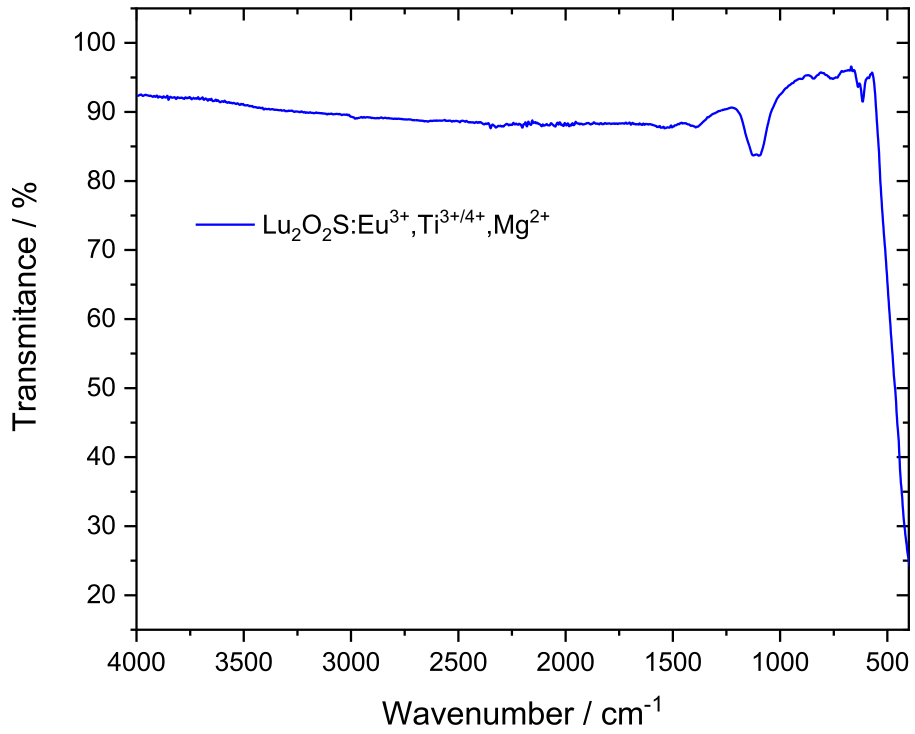

Figure S12. FTIR spectra of $Lu_2O_2S:Eu^{3+},Ti^{3+/4+},Mg^{2+}$ material.

3. DRS spectra

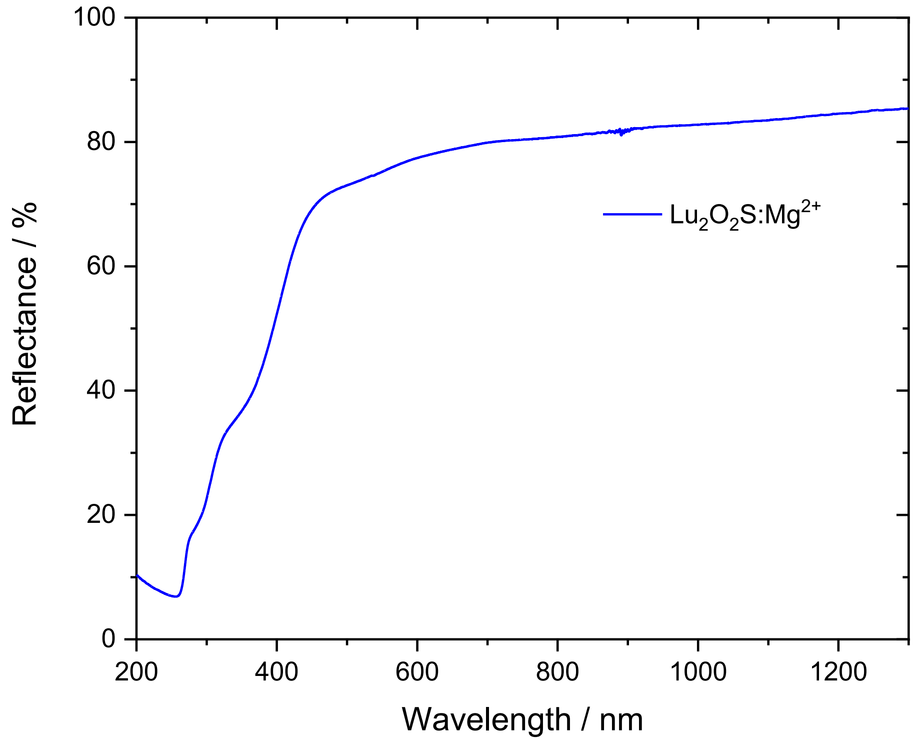

Figure S13. DRS spectra of $Lu_2O_2S:Mg^{2+}$ material.

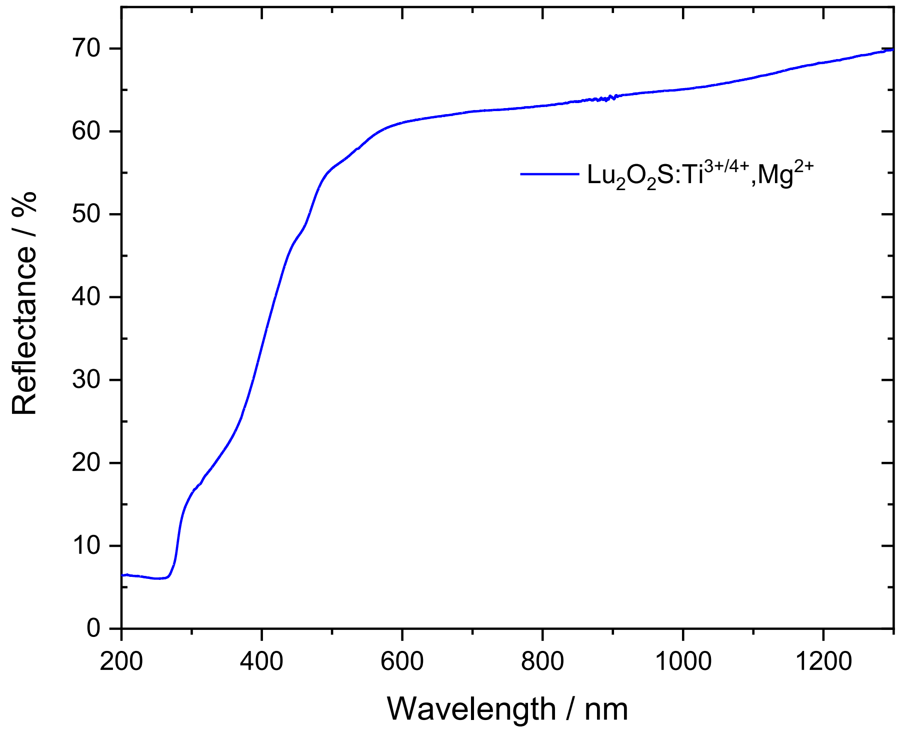

Figure S14. DRS spectra of Lu_2O_2S: $Ti^{3+/4+}$,Mg^{2+} material.

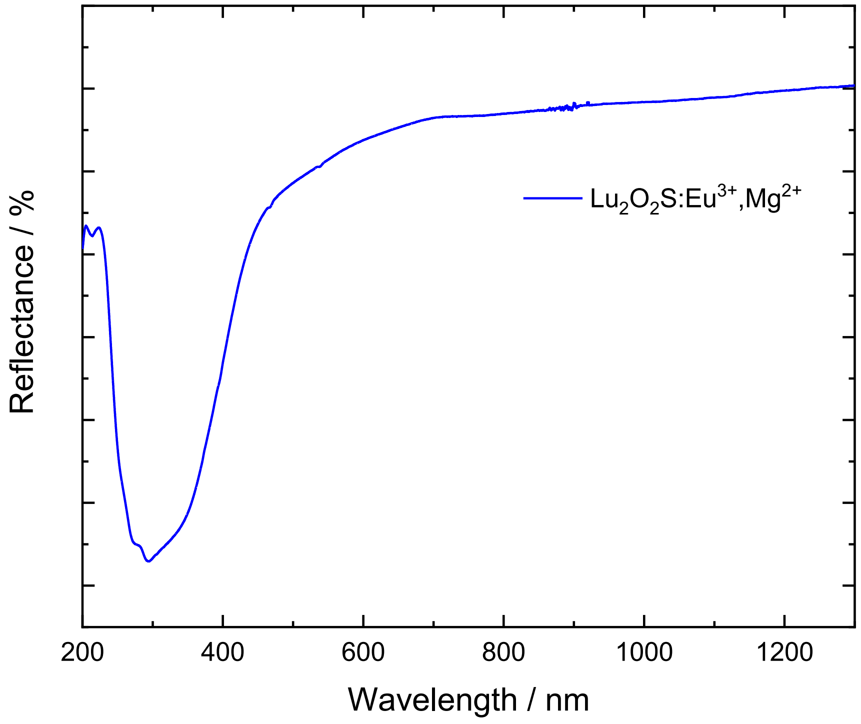

Figure S15. DRS spectra of $Lu_2O_2S:Eu^{3+},Mg^{2+}$ material.

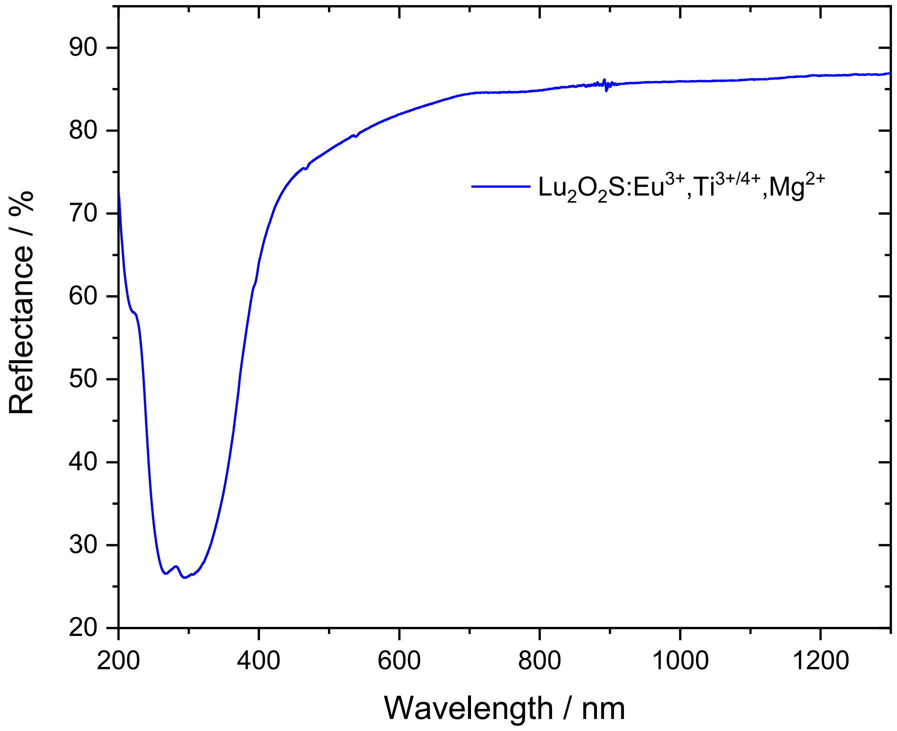

Figure S16. DRS spectra of $Lu_2O_2S:Eu^{3+},Ti^{3+/4+},Mg^{2+}$ material.

4. *EPR spectra*

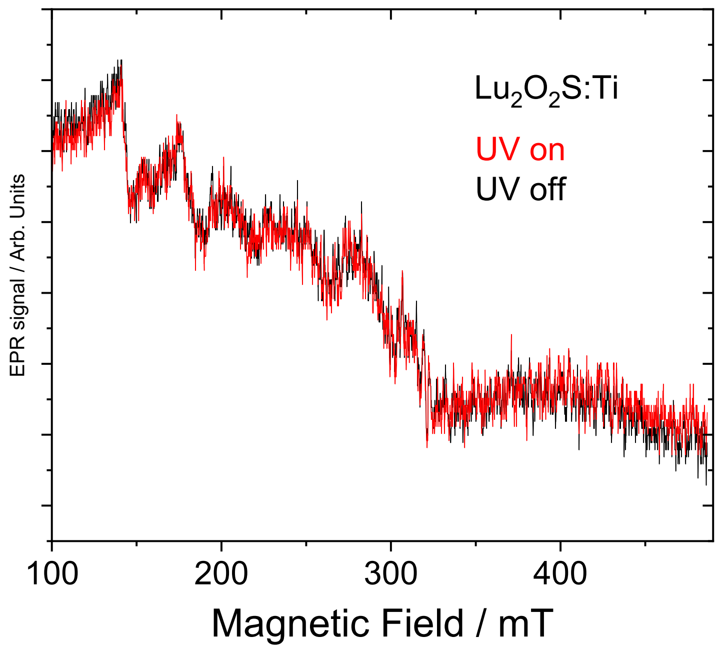

Figure S17. EPR spectra of Lu_2O_2S:Ti with and without UV irradiation.

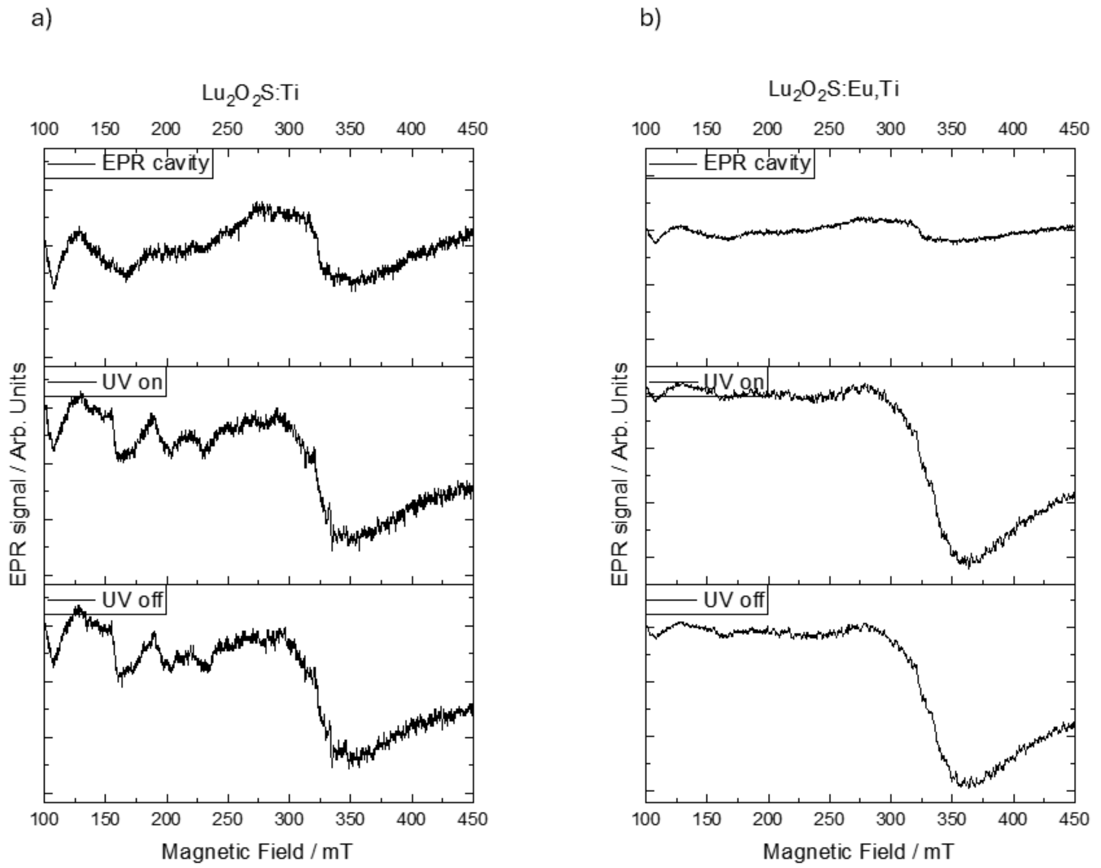

Figure S18. EPR spectra of Lu_2O_2S:Ti and Lu_2O_2S:Eu,Ti with and without UV irradiation. The empty cavity was also measured for comparison.

5. Experimental setup

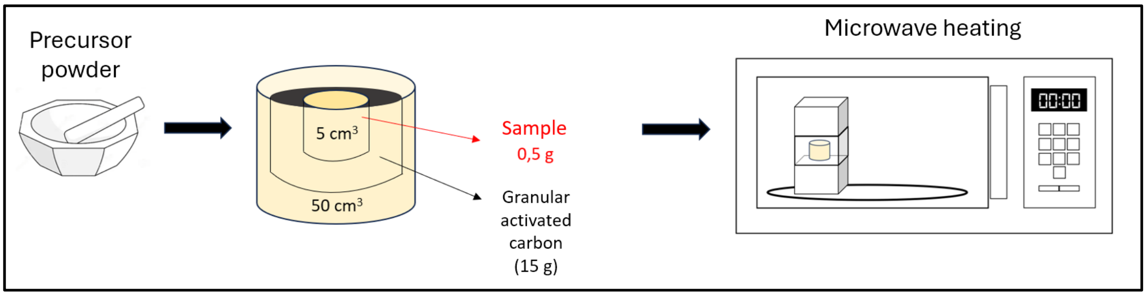

Figure S19. Schematic representation of the microwave-assisted solid-state synthesis method.

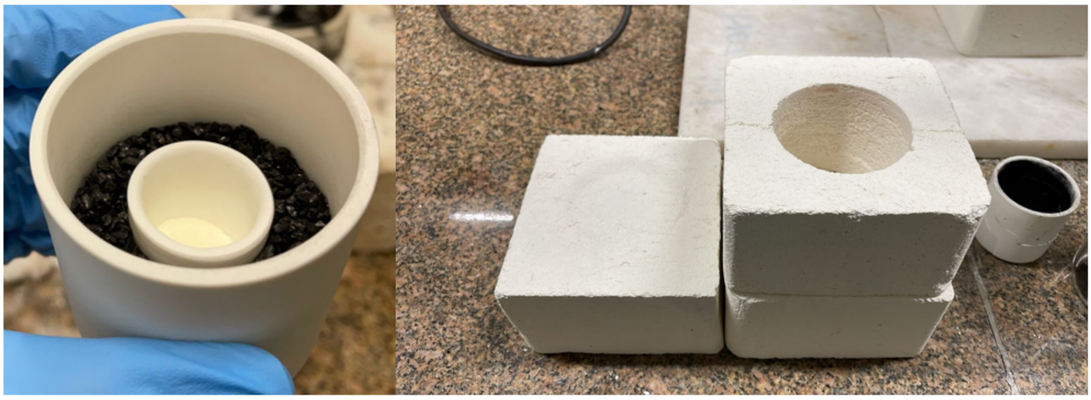

Figure S20. Key components of the MASS synthesis procedure.
